# Supplementary material for: The Hsp70-Hsp90 co-chaperone Hop/Stip1 shifts the proteostatic balance from folding towards degradation
Source: Nat Commun. 2020 Nov 25;11:5975. doi: 10.1038/s41467-020-19783-w (PMC7688965; doi:10.1038/s41467-020-19783-w)

Supplementary Information

The Hsp70-Hsp90 co-chaperone Hop/Stip1 shifts the proteostatic balance from folding towards degradation

Bhattacharya et al.

Supplementary Information includes Supplementary Methods, and twelve Supplementary Figures with legends.

Supplementary Methods

Induction of cellular stress and heat shock (HS). Human cells seeded at a density of 2 × 10^5 cells per 2 ml were treated with several stress inducers to evaluate the effect on cell death, apoptosis, and cellular morphology: 1 μM thapsigargin for 24 hrs, 20 μM A23187 for 24 hrs, 1 mM dithiothreitol (DTT) for 6 hrs, 1 mM H_2O_2 for 6 hrs, and 5 mM AZC (15 and 24 hrs for morphological and apoptosis analyses, respectively). To determine sensitivity to HS, cells seeded at a density of 4 × 10^5 per 2 ml were placed in a 43°C incubator for 0-60 min and allowed to recover in a 37°C incubator for 48 hrs. To assess HS-induced protein aggregation, cells seeded at a density of 4 × 10^6 per 10 ml were placed in a 43°C incubator for 0-120 min and immediately harvested to isolate the soluble and insoluble protein fractions. To assess the heat sensitivity of yeast cells, mutant and WT strains were grown in YPD medium at 30°C to mid-log phase (OD_{600} = 0.6-0.7). For spot tests, cells in liquid medium with an OD_{600} of 0.2 were spotted onto YPD agar plates, along with five spots of serial 5-fold dilutions. Plates were incubated at 37°C and 39°C for 3 days. Control plates were incubated at 30°C.

Pharmacological inhibition of biological processes. All experiments were performed with human WT and KO cells. To determine the effect of Hsp90 inhibition on the cell cycle and cell morphology, cells seeded at a density of 4 × 10^5 per 2 ml were treated with GA (0-1000 nM) or PU-H71 (0-1000 nM) for 24 hrs. For cell death analyses, cells seeded at a density of 4 × 10^5 per 2 ml were treated with GA (0-1000 nM) for 48 hrs and novobiocin (0-1000 μM) for 24 hrs. To assess the effect of GA on the aggregation of intracellular proteins, cells seeded at a density of 2 × 10^6 per 10 ml were treated for 24 hrs with 0-750 nM GA. To assess the effect of Hsp70 inhibition on cell cycle and cell morphology, cells seeded at a density of 4 × 10^5 per 2 ml were treated with JG-98 (0-2.5 μM) for 24 hrs. To assess the effect of Hsp90 inhibition on proteasomal activity and the stability of 19S RP-related proteins, cells seeded at a density of 2 × 10^6 per 10 ml were treated with GA (0-1000 nM) or JG-98 (0-5 μM) or both for 24 hrs. Cells seeded at a density of 2-4 × 10^5 per 2 ml were treated with proteasomal inhibitors MG132 and bortezomib for 24 hrs and 48 hrs, respectively, to determine the effect on cell death and cell morphology. To analyze the effect of the inhibition of ubiquitination on cell death and cell morphology, cells seeded at a density of 4 × 10^5 per 2 ml were treated with PYR41 (0-30 μM) for 48 hrs.

Proteotoxic challenge experiments were performed by treating cells seeded at a density of 4 × 10^5 per 2 ml with 500 nM GA for 6 hrs, followed by a 24 hrs treatment with either 10 μM MG132 or 500 nM bortezomib. Cell death was analyzed by flow cytometry. In other experiments, to analyze cell death, cells seeded at a density of 4 × 10^5 per 2 ml were heat-shocked at 43°C for 30-45 min before a 24 hrs treatment with MG132 (0-20 μM) at 37°C. To visualize the accumulation of ubiquitinated proteins during proteasomal inhibition, cells seeded at a density of 1.5 × 10^6 per 2 ml were treated with MG132 (0-10 μM) for 24 hrs and harvested for immunoblot analyses. Functional Hop rescue experiments were performed by transfecting KO cells seeded at a density of 5 × 10^5 per 2 ml with HA-tagged WT Hop. For these transfections, we used the Lipofectamine 3000 reagent according to the manufacturer's protocol. At 24 hrs post-transfection, cells were treated with MG132 (0-20 μM) or GA (0-1000 nM) for 24 hrs before the rescue effect on cell cycle and cell death was analyzed.

Protein expression and purification.

Hsp90α, Hsp90β, and mutants: Human Hsp90s were expressed and purified as described previously[2,3]. Hsp90s were expressed from the IPTG-inducible bacterial expression vector pCA528[4] as fusion proteins with an N-terminal 6× His-SUMO-tag in the *E. coli* strains BL21 (DE3) Star, pCodonPlus or Rosetta (DE3). Bacteria were harvested by centrifugation, resuspended in lysis buffer (40 mM HEPES-KOH pH 7.5, 100 mM KCl, 5 mM $MgCl_2$, 10% glycerol, 4 mM β-mercaptoethanol (β-ME), 5 mM PMSF, 1 mM pepstatin A, 1 mM aprotinin, 1 mM leupeptin) and lysed with a microfluidizer EmulsiFlex-C5 or a French press. Clarified lysates were incubated with a Ni-IDA affinity matrix and protein was eluted with lysis buffer containing 250 mM imidazole. To remove the SUMO-tag, Ulp1-SUMO protease was added to the eluted protein and dialyzed against lysis buffer containing 20 mM KCl overnight at 4°C. The dialyzed protein was loaded onto a Ni-IDA affinity matrix, followed by anion exchange chromatography (ResourceQ™; GE Healthcare Lifesciences) using a linear gradient of 0.02-1 M KCl. Eluted fractions of Hsp90 were further purified by size exclusion chromatography on a Superdex 200 (GE Healthcare Lifesciences) in storage buffer (40 mM HEPES-KOH pH 7.5, 50 mM KCl, 5 mM $MgCl_2$, 10% glycerol, 4 mM β-ME).

Hsp70 and mutant: WT human Hsp70 and the substrate binding mutant (V438F) were expressed in BL21 (DE3) Rosetta cells and purified as an N-terminal 6× His-SUMO fusion protein[3]. Bacterial cells were resuspended in lysis buffer (20 mM Tris-HCl pH 7.9, 100 mM KCl, 1 mM PMSF) and lysed with a microfluidizer EmusiFlex C5 or a French press. Clarified lysates were loaded onto Ni-IDA affinity matrixes. The affinity matrix was washed first with lysis buffer (without PMSF), then with high salt buffer (20 mM Tris-HCl pH 7.9, 1 M KCl), and again with lysis buffer. The affinity matrix was washed with ATP-buffer (40 mM Tris-HCl pH 7.9, 100 mM KCl, 5 mM $MgCl_2$, 5 mM ATP) to elute bound proteins. Hsp70 was eluted with 1 column volume of elution buffer (40 mM Tris-HCl pH 7.9, 100 mM KCl, 250 mM imidazole). Eluted proteins were dialyzed O/N against dialysis buffer (40 mM HEPES-KOH pH 7.6, 10 mM KCl, 5 mM $MgCl_2$) in the presence of Ulp1. The dialyzed sample was loaded onto a Ni-IDA affinity matrix and the flow-through containing the Hsp70 protein was subjected to a Resource™ Q anion exchange column. Hsp70 protein was eluted in elution buffer (40 mM HEPES-KOH pH 7.6, 1 M KCl, 5 mM $MgCl_2$, 10 mM β-ME, 5% glycerol) and further dialyzed against storage buffer (40 mM HEPES-KOH pH 7.6, 50 mM KCl, 5 mM $MgCl_2$, 10 mM β-ME, 10% glycerol).

Hop and Apg2: Hop and Apg2 proteins were expressed in BL21 (DE3) Rosetta cells (Merck KGaA, Darmstadt, Germany) and purified as an N-terminal 6× His-SUMO fusion protein as described previously[3]. Bacterial cells were resuspended in lysis buffer (40 mM Tris-HCl pH 7.9, 100 mM KCl, 5 mM ATP, 8 μg/ml pepstatin, 10 μg/ml aprotinin, 5 μg/ml leupeptin), and lysed with a microfluidizer EmulsiFlex-C5. The clarified lysates were loaded onto Ni-IDA affinity matrixes. Protein was eluted with buffer (40 mM Tris-HCl pH 7.9, 100 mM KCl) containing 300 mM imidazole. Subsequently, buffer exchange was performed using a HiPrep 26/10 Desalting Column (GE Healthcare Lifesciences). Ulp1-SUMO protease was added to the eluted protein and the solution was incubated O/N in the presence of 5 mM ATP. The protein was purified using a HiLoad 16/600 Superdex 200 gel filtration column (GE Healthcare

Lifesciences). Gel filtration eluents were further purified using anion-exchange chromatography on a Resource™ Q column (GE Healthcare Lifesciences) for Apg2, and POROS 20HQ column (Thermo Fisher Scientific) for Hop. Both proteins were eluted with a linear KCl gradient (0.01-1 M).

DnaJB1: Human DnaJB1 was expressed and purified as a 6× His-SUMO fusion protein as described previously[5].

Firefly luciferase: *Photinus pyralis* firefly luciferase was expressed and purified as described previously[6]. Firefly luciferase expressing XL10 Gold bacterial cells (Stratagene, US) were resuspended in lysis buffer (50 mM Na-phosphate pH 8.0, 300 mM NaCl, 10 mM β-ME, protease inhibitors, 10 μg/ml DNase) and lysed by microfluidizer EmulsiFlex-C5. Clarified lysate was applied to Ni-IDA affinity matrix beads and firefly luciferase was eluted with elution buffer (50 mM Na-phosphate pH 8.0, 300 mM NaCl, 250 mM Imidazole, 5 mM β-ME). Purified firefly luciferase was dialyzed O/N against dialysis buffer (50 mM Na-phosphate pH 8.0, 300 mM NaCl and 10 mM β-ME, 10% glycerol).

GST-UBL: GST-UBL was expressed and purified as described previously[7]. Expression of GST-UBL was induced using 0.1% (w/v) L-arabinose in the Rosetta (DE3) strain. Rosetta (DE3) cells were resuspended in GSH binding buffer (1× PBS, 10 mM $MgCl_2$, 1 mM DTT, PIC) and lysed with a French press. The clarified lysate was incubated with GSH-agarose affinity matrix and GST-UBL was eluted with an elution buffer (100 mM Tris-HCl pH 8, 100 mM NaCl, 1 mM DTT, 20 mM reduced GSH). Eluted protein was dialyzed twice against storage buffer (25 mM HEPES-KOH pH 7.4, 40 mM KCl, 5 mM $MgCl_2$, 10% glycerol, 1 mM DTT).

10×His-tagged-UIM: Expression of His-tagged-UIM was induced by IPTG in the Rosetta (DE3) bacterial strain. Bacterial cells were resuspended in lysis buffer (25 mM HEPES-KOH pH 7.4, 500 mM NaCl, 1 mM DTT, 0.025% NP-40, 20 mM imidazole) and lysed with a French press. The clarified lysate was incubated with Ni-NTA affinity matrix and His-tagged-UIM was eluted with an elution buffer (25 mM HEPES-KOH pH 7.4, 500 mM NaCl, 1 mM DTT, 0.025% NP-40, 500 mM imidazole). Eluted protein was dialyzed twice against the storage buffer[7].

Mass spectrometry.

Hsp90α and Hsp90β IP-MS sample preparation: Three biological replicates of WT and KO cells from HEK293T and HCT116 backgrounds were lysed in lysis buffer (20 mM HEPES-KOH pH 7.5, 50 mM KCl, 5 mM $MgCl_2$, 20 mM Na-molybdate, 1% NP40, protease inhibitors, phosphatase inhibitors). Clarified protein lysates (500 μg) were diluted in a total volume of 140 μl of incubation buffer (20 mM HEPES-KOH pH 7.5, 50 mM KCl, 5 mM $MgCl_2$, 20 mM Na-molybdate, 0.01% NP40). Hsp90 IPs were generated by incubating cell lysates with 15 μg of anti-Hsp90β and 20 μg anti-Hsp90α antibodies. Control IPs were performed with equal amounts of normal mouse IgG and normal rat IgG2a for 4 hrs at 4°C on a rotating wheel. Protein A-agarose (40 μl of a 50% slurry) was added to the IP mixtures and incubated for an additional 2 hrs. Beads were washed and proteins eluted with 60 μl FASP lysis buffer (100 mM Tris-HCl pH 7.5, 4% SDS, 10 mM TCEP) at 95°C for 5 min. The samples were digested by the

FASP method as described previously[8]. Resulting peptide mixtures were desalted on Waters SEP-PAK C18 micro elution plates and eluted with 100 µl of 40% acetonitrile, 0.1% formic acid. Eluates were separated into 6 fractions on SOLA SCX SPE columns (Thermo Fisher) using increasing concentrations of ammonium acetate (50, 75, 125, 250, 500, 1000 mM) in 20% acetonitrile and 0.5% formic acid. The dried peptides were resuspended in 25 µl solvent A (2% acetonitrile, 0.1% formic acid) and 5 µl samples were used for LC-MS/MS analysis.

Hop (HA) IP-MS sample preparation: Two biological replicates of HEK293T Hop KO cells were transfected with mammalian expression plasmids for WT or TPR double mutant (K8A, K229A) Hop. Empty vector transfected cells were used as a control. At 48 hrs post-transfection, cells were lysed by sonication in Hsp90 complex lysis buffer. Clarified cell lysates (2 mg) were incubated with 10 µg anti-HA antibodies O/N at 4ºC to IP exogenous HA-tagged WT or TPR double mutant Hop. Dynabeads™-Protein G were added to the incubations and proteins were eluted as described above. Eluted proteins were separated by 12% SDS-PAGE over a distance of 4 cm. After Coomassie blue staining, entire lanes were excised and underwent in-gel reduction and alkylation with chloroacetamide, before digestion with trypsin as described previously[9]. Extracted peptide mixtures were subjected to LC-MS/MS analysis.

Whole cell proteome MS sample preparation: Three biological replicates of WT and KO HEK293T and HCT116 cells were lysed and the proteins digested according to a modified version of the iST protocol[10]. Cells (5×10^6) were lysed in 250 µl modified iST buffer (100 mM Tris-HCl pH 8.6, 1% sodium deoxycholate, 10 mM DTT, protease inhibitors, phosphatase inhibitors) and heated at 95ºC for 5 min. The lysates were diluted 1:1 with 4 mM $MgCl_2$, benzonase nuclease was added and incubated for 15 min at RT. EDTA (3 mM) and 30 mM chloroacetamide were added for 45 min at 25ºC in the dark to alkylate cysteine residues. Samples were digested first with 2.5 µg of trypsin/Lys-C mix for 1 hr at 37ºC, followed by a second enzyme addition (1.25 µg trypsin/LysC) for 1 hr at 37ºC. To extract deoxycholate, 2 volumes of ethyl acetate and 1% TFA were added to 1 volume of lysate, the mixture was vortexed for 2 min and centrifuged. 100 µl of aqueous fractions were loaded onto equilibrated SOLA SCX SPE columns (Thermo Fisher) prefilled with 450 µl SCX0 buffer (20% acetonitrile, 0.5% formic acid) and centrifuged. The columns were washed once with 300 µl ethyl acetate, 0.5% TFA solution, and twice with 300 µl solvent A. The peptide mixtures were sequentially eluted with 200 µl SCX125 buffer (20% acetonitrile, 0.5% formic acid, 125 mM ammonium acetate), 200 µl SCX500 buffer (20% acetonitrile, 0.5% formic acid, 500 mM ammonium acetate), and finally with 200 µl basic elution buffer (80% acetonitrile, 19% H_2O, 0.25% NH_3). The dried fractions were resuspended in 100 µl solvent A and 5 µl solutions were used for LC-MS/MS analysis.

Purified proteasome MS sample preparation: Each sample preparation used 15-25 µg purified proteasomes. Chloroacetamide was added at the final concentration of 5 mM. The samples were diluted 1:1 with 8 M urea and incubated at RT for 30 min to alkylate cysteine residues. Endoproteinase LysC (0.2 µg) was added to the mixtures and the digestion was carried out for 2 hrs at 37ºC. The resulting solutions were diluted with 50 mM ammonium bicarbonate buffer and 0.5 µg of sequencing grade trypsin

(Promega) was added before the samples were incubated at 37ºC O/N. Digested samples were acidified with formic acid, and half of each digested sample was desalted on Waters C18 microelution plates by centrifugation. Peptides were eluted with 100 *µ*l of 40% acetonitrile and 0.1% formic acid solution. Dried samples were resuspended in 25 *µ*l 0.05% TFA for LC-MS/MS analysis.

References to Supplementary Methods

1. Guerrero, C., Milenkovic, T., Przulj, N., Kaiser, P. & Huang, L. Characterization of the proteasome interaction network using a QTAX-based tag-team strategy and protein interaction network analysis. *Proc. Natl. Acad. Sci. USA* **105**, 13333-13338 (2008).
2. Nguyen, M. T. N. et al. Isoform-specific phosphorylation in human Hsp90β affects interaction with clients and the cochaperone Cdc37. *J. Mol. Biol.* **429**, 732-752 (2017).
3. Morán Luengo, T., Kityk, R., Mayer, M. P. & Rüdiger, S. G. D. Hsp90 breaks the deadlock of the Hsp70 chaperone system. *Mol. Cell* **70**, 545-552 e549 (2018).
4. Andreasson, C., Fiaux, J., Rampelt, H., Mayer, M. P. & Bukau, B. Hsp110 is a nucleotide-activated exchange factor for Hsp70. *J. Biol. Chem.* **283**, 8877-8884 (2008).
5. Malakhov, M. P. et al. SUMO fusions and SUMO-specific protease for efficient expression and purification of proteins. *J. Struct. Funct. Genomics* **5**, 75-86 (2004).
6. Rampelt, H. et al. Metazoan Hsp70 machines use Hsp110 to power protein disaggregation. *EMBO J.* **31**, 4221-4235 (2012).
7. Besche, H. C. & Goldberg, A. L. Affinity purification of mammalian 26S proteasomes using an ubiquitin-like domain. *Methods Mol. Biol.* **832**, 423-432 (2012).
8. Wisniewski, J. R., Zougman, A., Nagaraj, N. & Mann, M. Universal sample preparation method for proteome analysis. *Nat. Methods* **6**, 359-362 (2009).
9. Wilm, M. et al. Femtomole sequencing of proteins from polyacrylamide gels by nano-electrospray mass spectrometry. *Nature* **379**, 466-469 (1996).
10. Kulak, N. A., Pichler, G., Paron, I., Nagaraj, N. & Mann, M. Minimal, encapsulated proteomic-sample processing applied to copy-number estimation in eukaryotic cells. *Nat. Methods* **11**, 319-324 (2014).

Supplementary Figure Legends

Supplementary Figure 1. KO cells maintain proteostasis and are not hyper-sensitive to proteotoxic stresses, related to Fig. 1.
a Quantitation of the *STIP1* mRNA of WT and Hop KO clones by Q-PCR using the two different primer pairs STIP1_1 and STIP1_2 for the 5' and 3' regions, respectively, of the *STIP1* mRNA (n = 2 biologically independent samples). **b** Phase contrast micrographs of Hop WT and KO cells. **c** Cell proliferation analysis with an MTT assay (n = 3 biologically independent samples). **d** Flow cytometry histograms representing the cell cycle distributions of WT and KO cells. **e** Dot plots representing live/dead cell distributions visualized by annexin V-FITC (AnnxV-FITC) and PI staining using flow cytometry; the number in each quadrant gives the % of the total cell population. **f** Phase contrast micrographs of WT and KO cells treated with DTT or H_2O_2; vehicle-treated cells serve as a control. **g** Flow cytometric quantification of apoptotic cells after 48 hrs of recovery at 37°C following a HS for 0 to 60 min (n = 4 biologically independent samples). **h** Phase contrast micrographs of WT and KO cells treated with AZC; vehicle-treated cells serve as a control. HEK, HEK293T; HCT, HCT116. For panels **b, f, and h**, a representative scale bar is shown on one of the micrographs of each panel and indicates 100 μm. For the bar graphs, the data are represented as mean values ± SEM. The statistical significance between the groups was analyzed by two-tail unpaired Student's t-test. Source data are provided as a Source Data file.

Supplementary Figure 2. Inhibition of Hsp70 and Hsp90 or the proteasome have differential impacts on KO cells, related to Fig. 2.
a Flow cytometric analysis of the GA-induced G2/M phase cell cycle arrest with A549 cells treated with GA for 24 hrs (n = 2 biologically independent samples). % G2/M phase arrest was calculated as indicated in the legend to Fig. 2b. Data are represented as a box plot. **b** Flow cytometry histograms representing the impact of a treatment with GA for 48 hrs. The number over the linear gate in each plot indicates the % PI positive dead cells in the total analyzed cell population. **c** Flow cytometric analysis of the PU-H71-induced G2/M phase cell cycle arrest (left) and apoptosis (right) after 24 hrs of treatment (n = 2 biologically independent samples). % G2/M phase arrest and % Apoptosis was calculated as indicated in the legend to Fig. 2b. Data are represented as a box plot. **d** Flow cytometric analysis of cell death induced by novobiocin (Novo) after 24 hrs of treatment (n = 3 biologically independent samples). **e** Phase contrast micrographs of WT and KO cells treated with JG-98 for 24 hrs. Vehicle-treated cells serve as a control. A representative scale bar indicating 100 μm is shown in one of the micrographs. **f, g** Flow cytometric analysis of MG132- and bortezomib-induced cell death after 24 and 48 hrs of treatment, respectively (n = 3 biologically independent samples). **h** Flow cytometric analysis of the PYR41-induced cell death (n = 3 biologically independent samples). **i** Immunoblot assays to determine the protein translation rate based on the incorporation of puromycin into nascent polypeptide chains. For the bar graphs, the data are represented as mean values ± SEM. For box plots, data are represented as the median values and edges of the box plots represent the range of the data. The statistical significance between the groups was analyzed by two-tail unpaired Student's t-test. Source data are provided as a Source Data file.

Supplementary Figure 3. Interactions of Hop with proteasomal subunits, related to Fig. 3.

a IP of HA-tagged WT and TPR mutants of Hop overexpressed in HEK293T KO1 cells with an anti-HA antibody, visualized by Coomassie blue staining of the SDS-PAGE. **b** Anti-HA immunoprecipitations of WT and TPR double mutant (K8A, K229A) HA-tagged Hop overexpressed in HEK293T KO1 cells; cells transfected with empty vector were used as negative control. Two biological replicates for each sample were visualized by Coomassie staining following SDS-PAGE. Each lane was processed for LC-MS/MS analysis to identify Hop interactors. **c-e** GO term enrichment analyses of identified Hop interactors (see also Fig. 3a). GO term enrichment analyses were done with the Enricher web server, and the top 10 KEGG and WIKI pathway-annotated biological processes are plotted according to the P values (panel **c**), % overlap of MS-identified proteins with annotated proteins having a known GO function (panel **d**), and a combined score provided by the Enricher web server (panel **e**). Proteasomal and ubiquitin-related pathways are indicated with red arrows. **f** Interaction matrix of components of the ternary molecular chaperone complex identified as interactors of proteasome components purified from yeast. Data were obtained from a previously published MS dataset of the affinity-purified proteasome[1]. Source data are provided as a Source Data file.

Supplementary Figure 4. The absence of the Hsp70-Hop-Hsp90 ternary complex compromises proteasomal function in KO cells, related to Fig. 4.

a *In vitro* steady-state proteasomal activity of extracts of WT and KO HCT116 (n = 4 biologically independent samples) and A549 (n = 6 biologically independent samples) cells determined with the activity reporter suc-LLVY-AMC. **b** Rate of proteasomal activity determined with the activity reporter suc-LLVY-AMC. n = 4 biologically independent samples for both cell lines. **c** Flow cytometric determination of the *in vivo* UPS activity using the Ub-M-GFP and Ub-R-GFP reporter plasmids (n = 3 biologically independent samples). **d** Immunoblots of overexpressed HA-tagged WT and TPR mutants of Hop. A representative immunoblot analysis for the quality control of cell extracts corresponding to those assayed for proteasomal activity in Fig. 4d. **e** Flow cytometric measurement of autophagic flux using a mCherry-GFP-LC3 reporter. It is calculated as the ratio of the mean fluorescence intensities of GFP and mCherry positive cells. n = 6 and 4 biologically independent samples for HEK293T and HCT116, respectively. **f** *In vitro* steady-state proteasomal activity of WT, and Hsp90α and Hsp90β KO cells determined with the activity reporter suc-LLVY-AMC (n = 3 biologically independent samples). Representative immunoblots confirm the absence of Hsp90α and Hsp90β, respectively. **g** Impact of GA (left, n = 3 biologically independent samples) and JG-98 (middle, n = 2 biologically independent samples) alone, and in combination (right, n = 3 biologically independent samples) on the proteasomal activity of extracts from WT HEK293T cells treated for 24 hrs. Activity was measured 20 and 40 min after the initiation of the reaction with suc-LLVY-AMC. The activity of untreated controls was set to 100%. **h** Impacts of GA and MG132 on the proteasomal activity of an extract of HEK293T WT cells measured by *in vitro* proteasomal activity assay (n = 2 biologically independent samples). Proteasomal activities were measured at 20 min and 40 min after initiation of the reaction with suc-LLVY-AMC. Data are represented as total AMC fluorescence, and MG132 serves as a positive control for proteasomal inhibition. **i** Rate of proteasomal activity of extracts

from WT and *Δsti1* yeast cells (BY4741 strain background) using activity reporter suc-LLVY-AMC corresponding to the steady-state proteasomal activity assay shown in Fig. 4e. n = 5 and 4 biologically independent samples for overnight (O/N) and Mid-Log cultures, respectively. For the bar graphs and line graphs, the data are represented as mean values ± SEM. The statistical significance between the groups was analyzed by two-tail unpaired Student's t-test. Source data are provided as a Source Data file.

Supplementary Figure 5. Characterization of the 26S/30S proteasome purified from Hop WT and KO cells, related to Fig. 5.
a, b Visualization of the purified 26S/30S proteasome by 4% native-PAGE. Immunoblots were probed with antibodies to Psmd2 or Psmc5 for the RP, and to Psma3 for the CP. Ponceau S stained nitrocellulose filters serve as controls of equal loading of purified proteasomes. **c, d** The activity of the purified proteasomes determined with activity reporter suc-LLVY-AMC (n = 2 independent experiments). Data are represented as total AMC fluorescence, and MG132 serves as a positive control for proteasomal inhibition. Data are represented as a box plot. **e, f** Abundance (log2 of iBAQ values) of all proteasomal proteins identified by the MS analysis of purified proteasomes. Stoichiometric RP- and CP-specific proteasomal proteins are in green and blue, respectively. For the bar graphs, the data are represented as mean values. For box plots, data are represented as the median values and edges of the box plots represent the range of the data. Source data are provided as a Source Data file and supplementary data file.

Supplementary Figure 6. The Hsp70-Hop-Hsp90 ternary complex facilitates assembly of the proteasome, related to Fig. 5.
a Abundance of different proteasomal particles of A549 Hop WT and KO cells displayed by 4% native-PAGE and subsequent immunoblotting as in Fig. 5f (n = 4 independent samples over 2 independent experiments). Positions of 26S/30S proteasome particles and free 20S CP are indicated using the bands of purified proteasome particles as standards. The two different exposures are from the same immunoblot. **b** Representative immunoblots of Psma3 and Hop in WT and KO cell lysates, which were used for the native-PAGE analysis of Fig. 5f and Supplementary Figure 6a (n = 2 independent experiments). **c** Abundance of different proteasomal complexes determined by 4% native-PAGE. Note that 1.5-fold more cell lysate (Ly) of KO than of WT cells was loaded for this experiment (n = 1 along with similar results with n = 5 independent samples over 3 independent experiments shown in Fig. 5f). Antibodies to Psma3 were used to probe for proteasomal complexes. L.E. and S.E., long and short exposures, respectively. **d** Native-PAGE and subsequent immunoblot analyses of extracts from KO cells overexpressing exogenous WT and TPR double mutant (K8A, K229A) Hop (n = 2 independent experiments). Antibodies to Psmc5 and Psma3 were used to probe for proteasomal complexes. An extract of KO cells transfected with empty vector was used as a negative control. **e** Control immunoblot analyses of the same cell extracts of panel **d** (n = 2 independent experiments). **f** Native-PAGE and subsequent immunoblot analyses of extracts from mid-log yeast cells (n = 4 independent samples over 2 independent experiments). Antibodies to Psma3 were used to probe for proteasomal complexes. Positions of 26S/30S proteasome particles and free 20S CP are indicated using the bands of purified proteasome particles as standards. **g** Immunoblots of Sti1 and indicated proteasomal

components in WT and *Δsti1* yeast cells (n = 2 independent experiments). **h** Control immunoblot analyses of the antibodies that were used for the immunofluorescence experiment shown in panel **i** (n = 2 independent experiments). **i** Immunofluorescence analyses of the indicated proteasomal subunits. DNA was stained with DAPI (blue) (n = 2 independent biological samples). Scale bar represents 10 μm.

Supplementary Figure 7. Proteasomal utilization is reduced in KO cells, even in proteotoxic stress, related to Fig. 5.
a Nitrocellulose filter stained with Ponceau S displaying the GA-induced SDS-soluble protein aggregates. **b** Immunoblot of the insoluble ubiquitinated proteins after a 43°C HS. The loaded SDS-soluble material is from equal numbers of cells. **c-e** Flow cytometric quantification of cell death induced by MG132 (10 μM) and bortezomib (Bortz, 500 nM) alone and in combination with a 6 hr pretreatment with GA (500 nM); number of biologically independent samples: n = 3, n = 2, and n = 3 for panels **c**, **d**, and **e**, respectively. Below panel **d**, scheme of the experiment in red. **f, g** Flow cytometric quantification of cell death induced by treatment with MG132 for 24 hrs during the recovery phase at 37°C of a HS (43°C, 30 or 45 min, n = 3 biologically independent samples). Schemes of the experiment in red. See Methods for how the Y axis values were calculated and for why there can be negative values. **h** Immunoblot of ubiquitinated proteins in total SDS-soluble lysates. For the bar graphs, the data are represented as mean values ± SEM. Source data are provided as a Source Data file.

Supplementary Figure 8. Hop-independent enhanced chaperoning by Hsp70 and Hsp90 in human and yeast cells, related to Fig. 6.
a Immunoblot of ubiquitinated proteins from soluble and insoluble protein fractions. **b** *In vivo* refolding of heat-denatured luciferase (n = 3 biologically independent samples). **c** *In vivo* luciferase refolding in WT HEK293T cells treated with GA (1 μM) and JG-98 (2 μM) (n = 2 biologically independent samples) starting 1 hr prior to HS and during the recovery phase at 37°C. **d** Growth assays with WT and *Δsti1* yeast cells of the W303 strain background. Spot tests of serial 5-fold dilutions; cells grown at indicated temperatures for 3 days. **e** *In vivo* refolding of heat-denatured luciferase in WT and *Δsti1* yeast cells of the W303 strain background during the recovery phase at 30°C (n = at least 8 biologically independent samples). **f** Growth assays with yeast cells of the BY4741 strain background as in panel **d**. **g** Fluorescence micrographs of cells expressing the fusion proteins Q74-EGFP and Q23-EGFP. Q74-EGFP aggregates are visible as punctate green fluorescence. Aggregates appear as dark black dots marked with red circles in the inverted grayscale images. The non-aggregating Q23-EGFP serves as a negative control. The scale bar in one of the micrographs indicates 50 μm and is the same for all micrographs. **h** Solubility of aggregation-prone polyglutamine model protein Q74-EGFP in WT and KO HCT116 cells. Immunoblots as in Fig. 6f. For the line graphs, the data are represented as mean values ± SEM. The statistical significance between the groups was analyzed by two-tail unpaired Student's t-test. Source data are provided as a Source Data file.

Supplementary Figure 9. Hsp90 client-specific impact of the Hop KO, related to Fig. 7.
a Heat maps of the normalized fold changes of the levels of Hsp90 clients (left), and molecular chaperones and co-chaperones (right) identified by whole cell MS analyses.

The scale bar represents the log2 fold changes (WT vs KO) of the LFQ values. **b** Immunoblots of total cellular proteins phosphorylated on either tyrosine or serine. Note that the left panel is an overexposure of the lanes labelled "Mock" in the middle of Supplementary Fig. 9g. **c** Immunoblots of phospho- and total c-Src (left part) and Erk1/2 (right part). Nitrocellulose filters stained with Ponceau S indicate equal protein loading. **d** Transcriptional activities of Hsp90 clients determined with corresponding luciferase reporter genes. Data are represented as fold change of transcriptional activities in KO cells in comparison with WT cells (set at 1, n = 3 biologically independent samples). NFkB, nuclear factor kappa B; CFOS, c-Fos; CTNNB1, β-catenin; TP53, WT p53; HIF, hypoxia-inducible factor; HSF, heat-shock factor. **e** Immunoblots of F-GR overexpressed in HCT116 cells. Tf, transfected cells. **f, g** Accumulation and activity of overexpressed v-Src in the indicated cellular backgrounds. Tyrosine-phosphorylated total protein is indicative of v-Src activity. The nitrocellulose filter stained with Ponceau S serves as a loading control. Tf, transfected cells. **h** Immunoblots of overexpressed HA-tagged Hif-1α and Hif-2α, and of androgen receptor (AR). EGFP serves as a transfection control. For the bar graph, the data are represented as mean values ± SEM. Source data are provided as a Source Data file.

Supplementary Figure 10. Human Hsp70 and Hsp90 interact directly and form a functional prokaryote-like molecular chaperone complex even in the absence of Hop, related to Fig. 8 and 9.
a Abundance of the top 20 Hsp90 interactors (according to the highest iBAQ values) of KO HEK293T cells (names in bold). The graph shows the iBAQ values as log2 of the Hsp90 IP-MS analyses. Hsp90α (HSP90AA1) and Hsp90β (HSP90AB1) were the bait proteins, and absence of Hop (STIP1) serves as quality control marker for KO cells (n = 3 biologically independent samples). Inset: Linear fold changes of the values for Hsp70 (HSPA1) and Hsc70 (HSPA8). *Ub proteins: UBB, UBC, UBA52, RPS27A. **b** *In vivo* interaction of Hsp90 and Hsp70 in A549 cells as determined by an immunoprecipitation experiment. **c** Bar graph showing the enrichment (green bars) or depletion (grey bars) of multiple TPR domain-containing proteins identified in the Hsp90 IP-MS analyses (n = 3 biologically independent samples). **d** Relative abundance of the multiple TPR domain-containing proteins compared to Hsc70 (HSP8) and Hsp70 (HSPA1). The abundance of both Hsp70 and Hsc70 is indicated by red dotted lines. The graph shows the iBAQ values as log2 of the Hsp90 IP-MS analyses (n = 3 biologically independent samples). **e** Surface accessibility of the highlighted amino acids in the dimeric structure of HtpG in its open conformation. Color code as in Fig. 8f. **f** Interaction of Hsp90 point mutants with endogenous Hsc70 (n = 2 independent experiments). Immunoprecipitation of exogenously overexpressed FLAG-tagged WT and point mutant Hsp90α from KO33 cells. Immunoblots were probed with antibodies to FLAG or Hsc70. **g** The volcano plot represents the normalized fold changes of the Hsp90 interactors identified by the Hsp90 IP-MS analysis with HCT116 cells versus their VIP values derived from the OPLS-DA model. Cut-offs and color as in Fig. 9a. **h** Immunoblots showing time-dependent HS-induced recruitment of Hsp90, Hsp70, Hsp40 and Hsp110 into insoluble protein aggregates. The soluble protein fractions show the total levels of the aforementioned proteins. GAPDH serves as a control for soluble proteins even following HS. The loaded material is from equal numbers of cells. HS was at 43°C for the indicated time points. Note that the weak bands of the 120 min time points in the Hop immunoblots are non-

specific. For the bar graphs, the data are represented as mean values. Source data are provided as a Source Data file and a supplementary data file.

Supplementary Figure 11. Schematic representation of the FACS strategies for cell death and cell cycle analyses.
a FACS gating and analysis strategy for the propidium iodide (PI) positive population of dead cells; related to Figs. 1d, and 2d, e, h, and Supplementary Figs. 2b, d, f, g, and 7c-g. **b** FACS gating and analysis strategy of cell cycle experiments; related to Fig. 1e, f, and 2b, c, f, g, and Supplementary Figs. 1d, g, and 2a, c. **c** FACS gating and analysis strategy for cells subjected to dual staining with annexin V and PI; related to Supplementary Fig. 1e.

Supplementary Figure 12. Schematic representation of the FACS strategies for autophagic and *in vivo* UPS activities.
a FACS gating and analysis strategy for the autophagic flux analysis; related to Supplementary Fig. 4e. **b** FACS gating and analysis strategy of *in vivo* UPS activity measurements; related to Fig.4c and Supplementary Fig. 4c.

Supplementary Figure 1

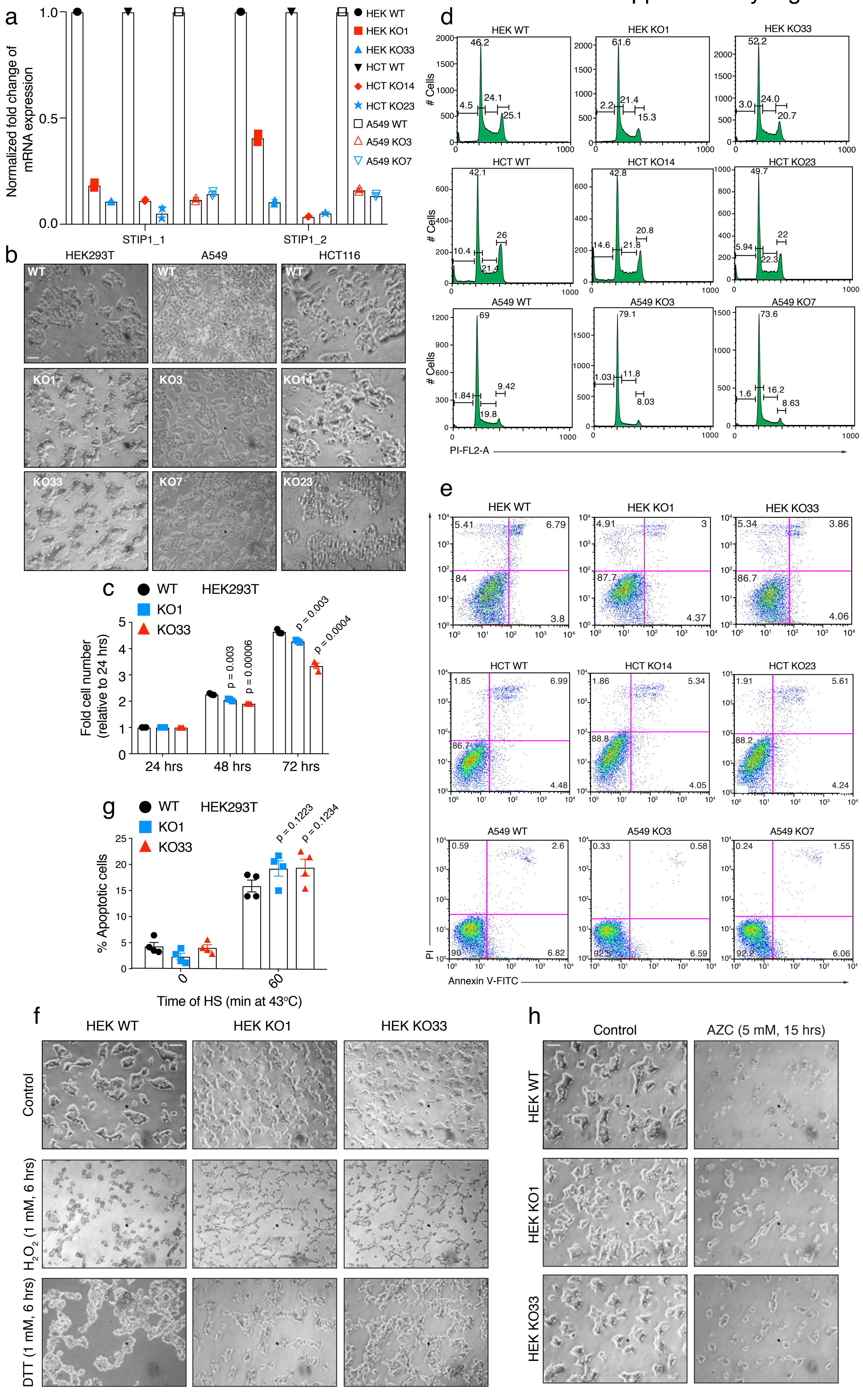

Supplementary Figure 2

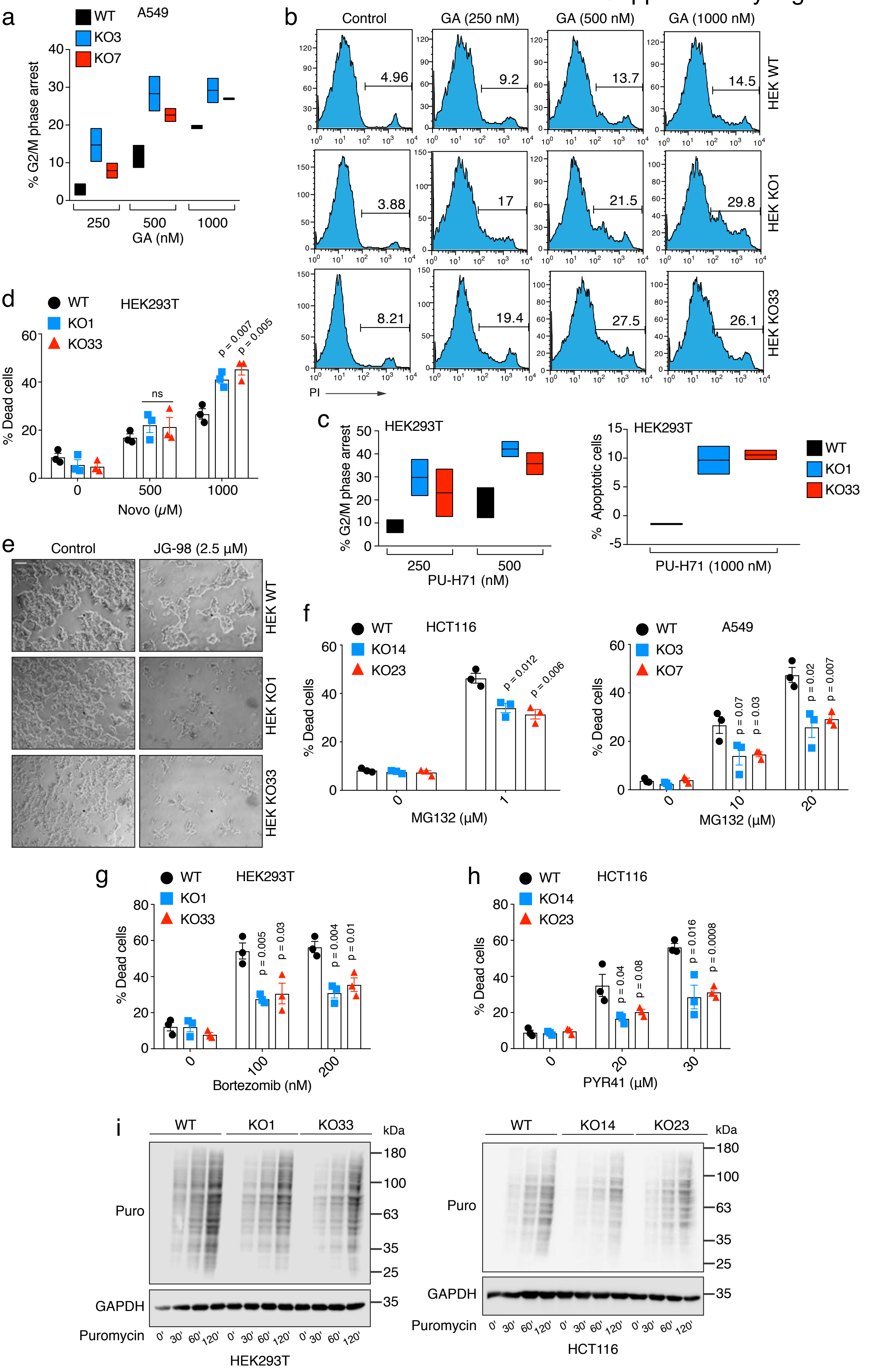

Supplementary Figure 3

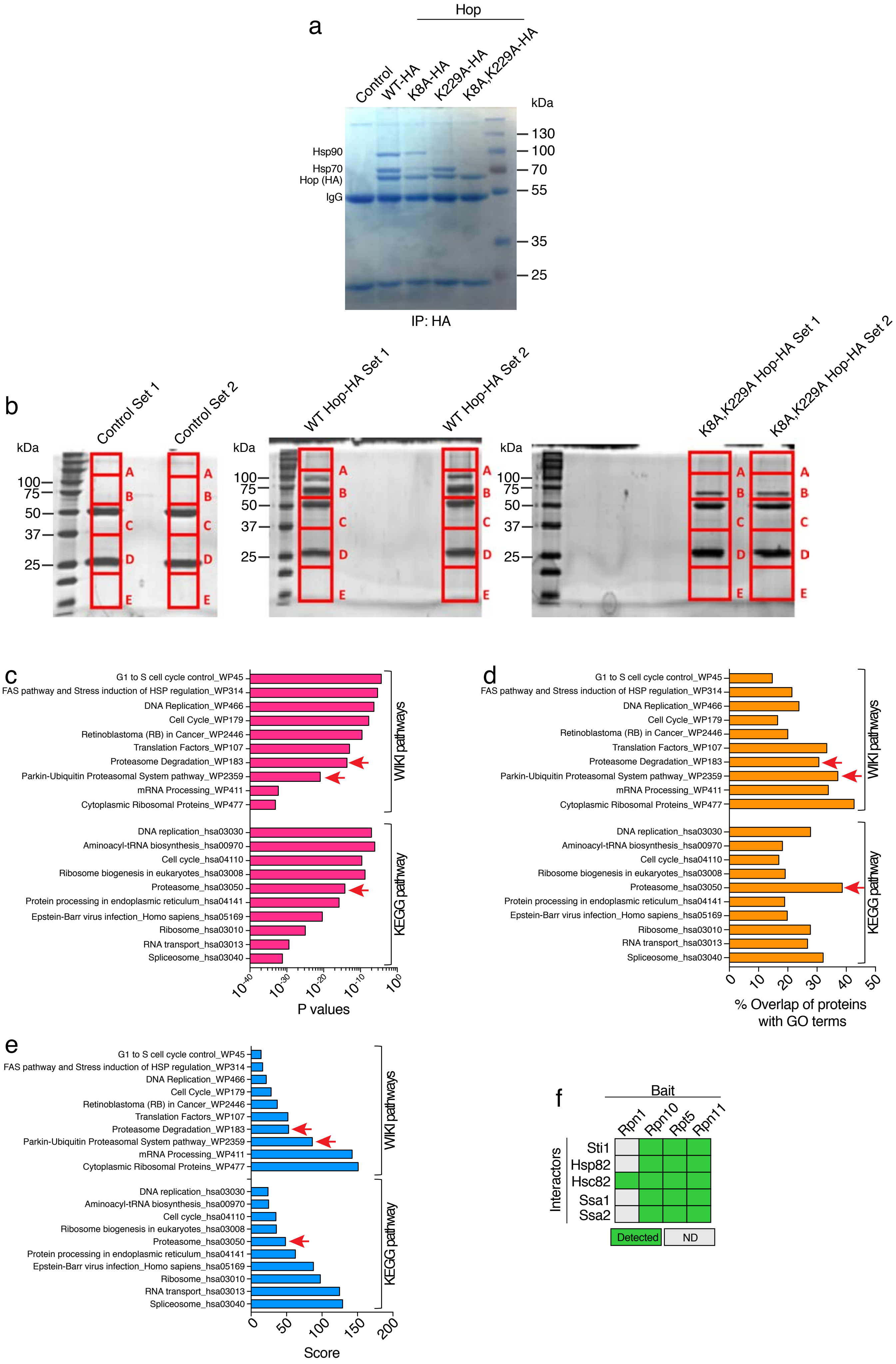

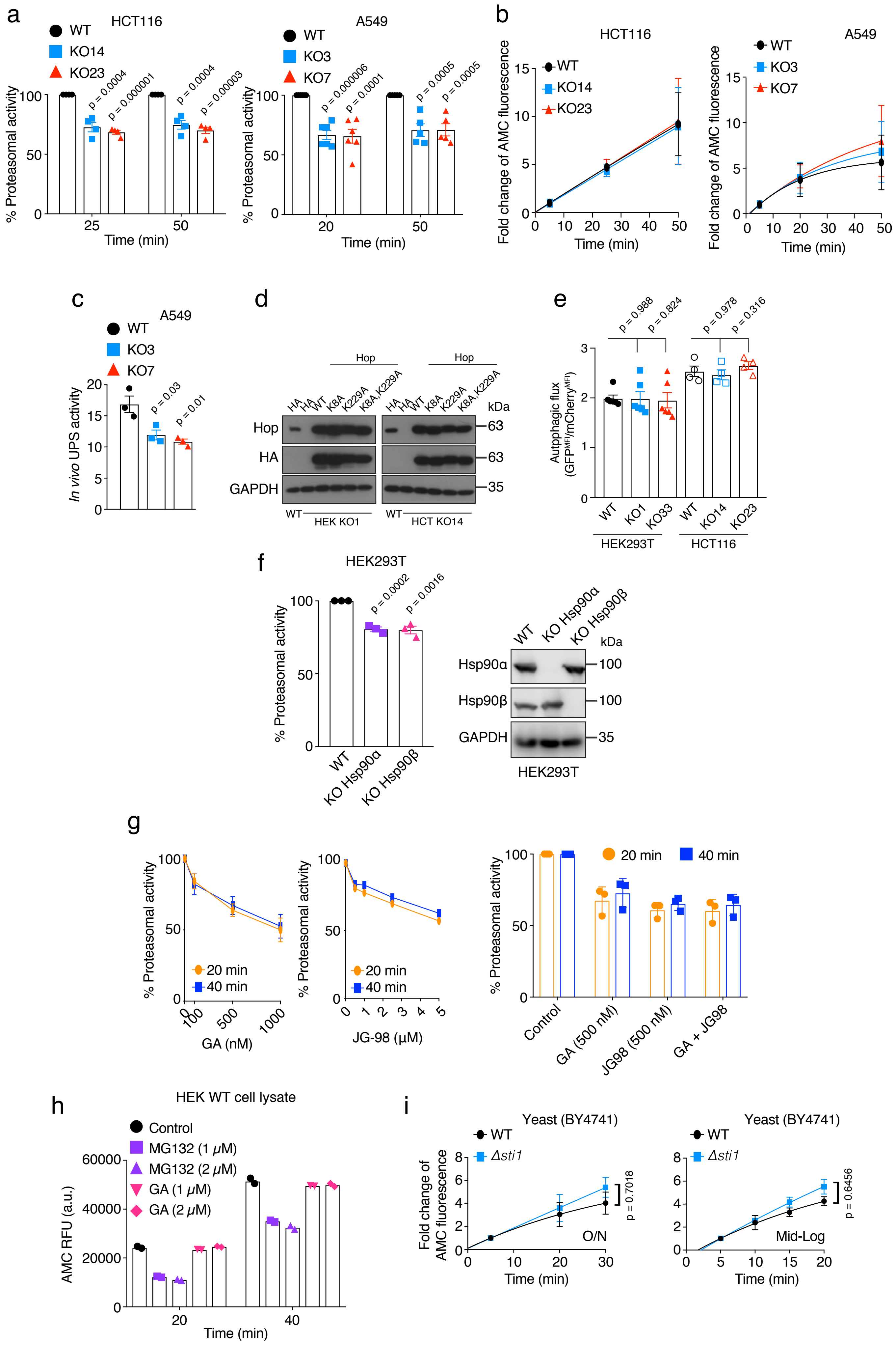
a
HCT116
WT
KO14
KO23
% Proteasomal activity
Time (min)
A549
WT
KO3
KO7
b
HCT116
Fold change of AMC fluorescence
A549
c
A549
In vivo UPS activity
d
Hop
HA
GAPDH
kDa
WT
HEK KO1
HCT KO14
e
Autphagic flux (GFP^MFI/mCherry^MFI)
HEK293T
HCT116
f
HEK293T
KO Hsp90α
KO Hsp90β
Hsp90α
Hsp90β
g
GA (nM)
JG-98 (µM)
20 min
40 min
Control
GA (500 nM)
JG98 (500 nM)
GA + JG98
h
HEK WT cell lysate
Control
MG132 (1 µM)
MG132 (2 µM)
GA (1 µM)
GA (2 µM)
AMC RFU (a.u.)
i
Yeast (BY4741)
Δsti1
Fold change of AMC fluorescence
O/N
Mid-Log
p = 0.7018
p = 0.6456

Supplementary Figure 5

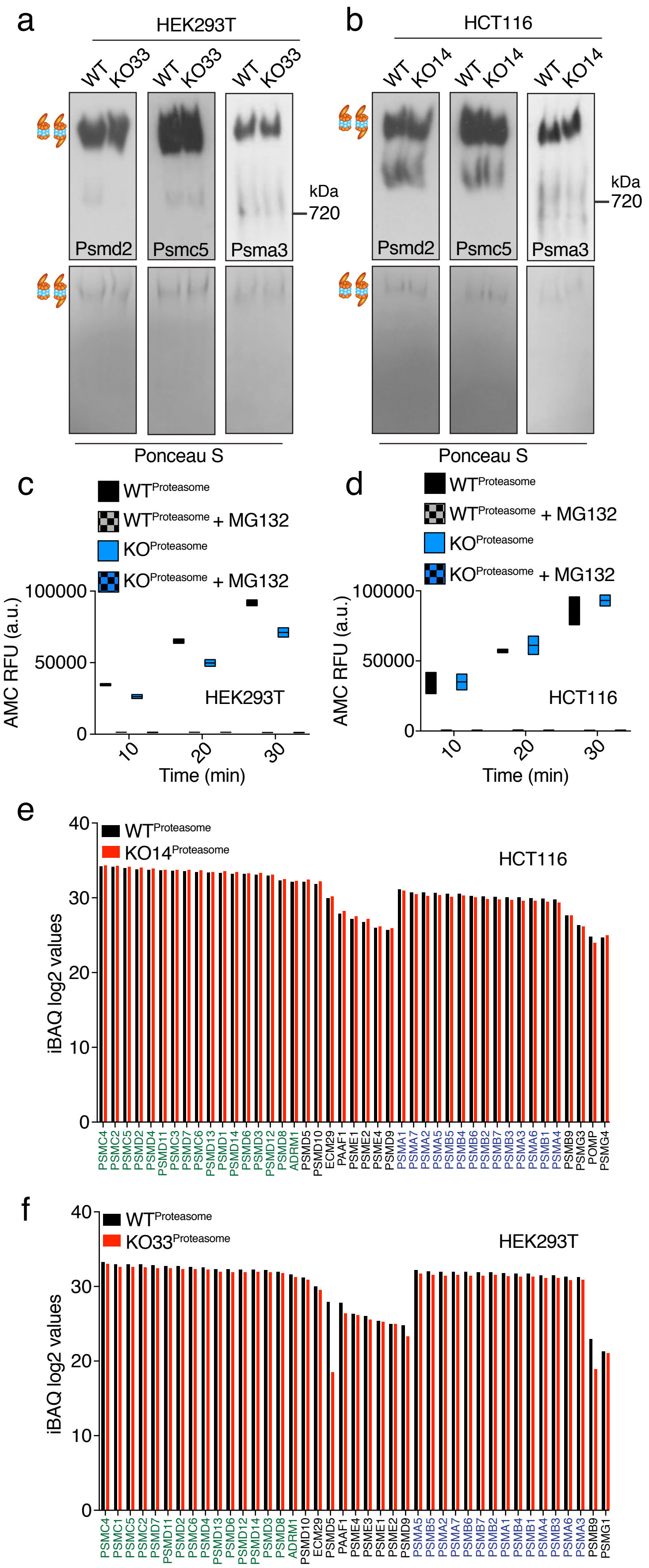

Supplementary Figure 6

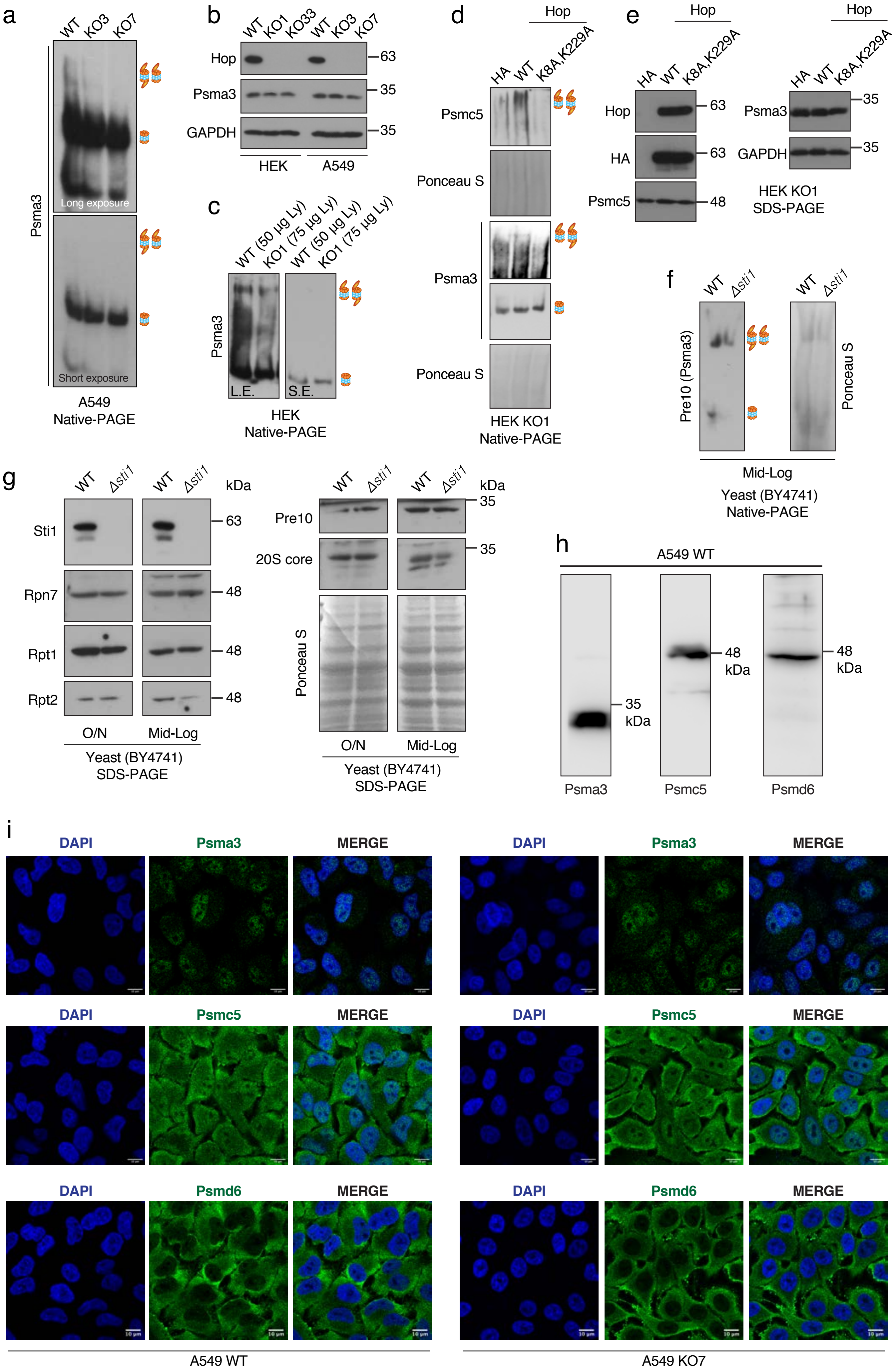

Supplementary Figure 7

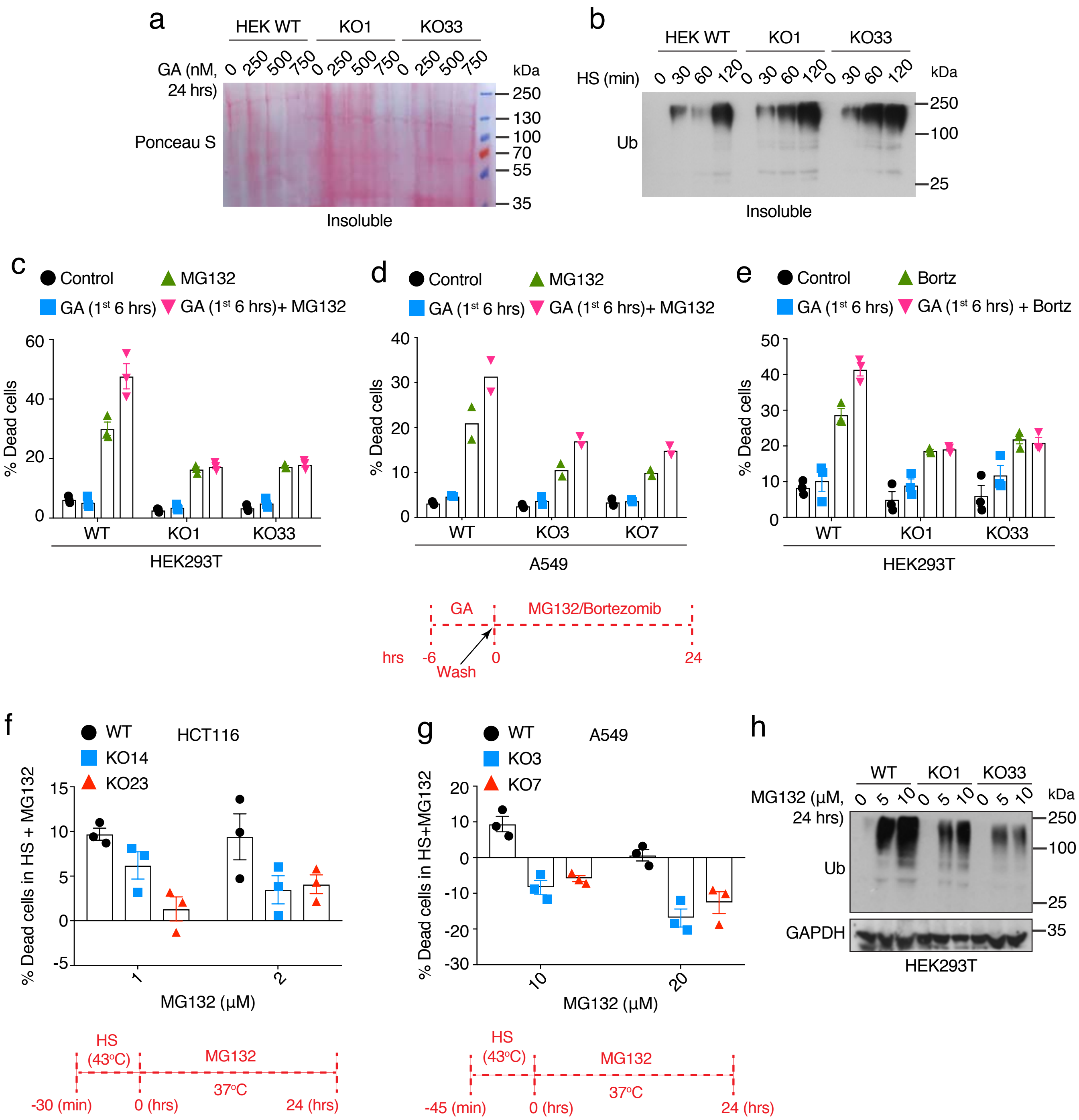

Supplementary Figure 8

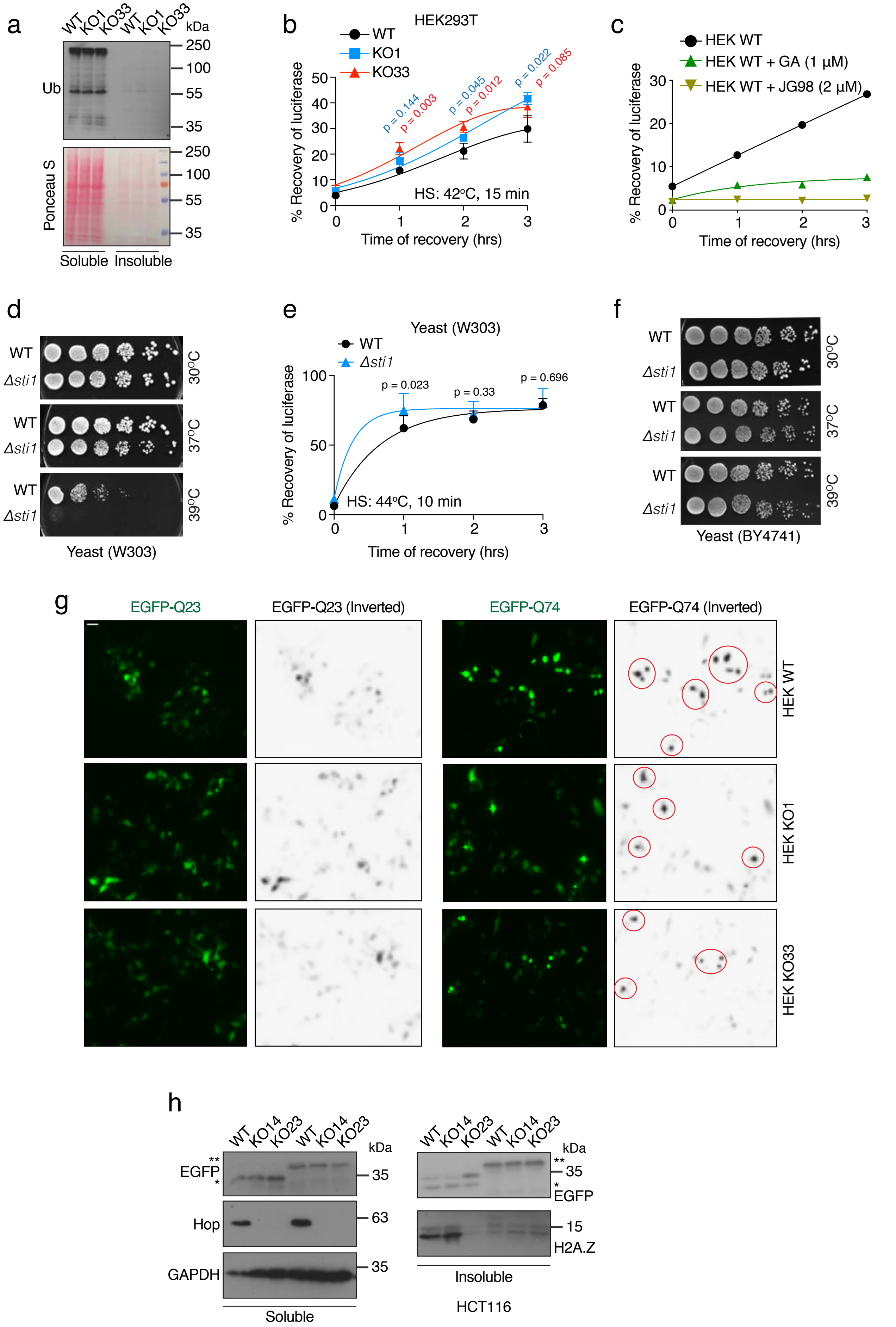

Supplementary Figure 9

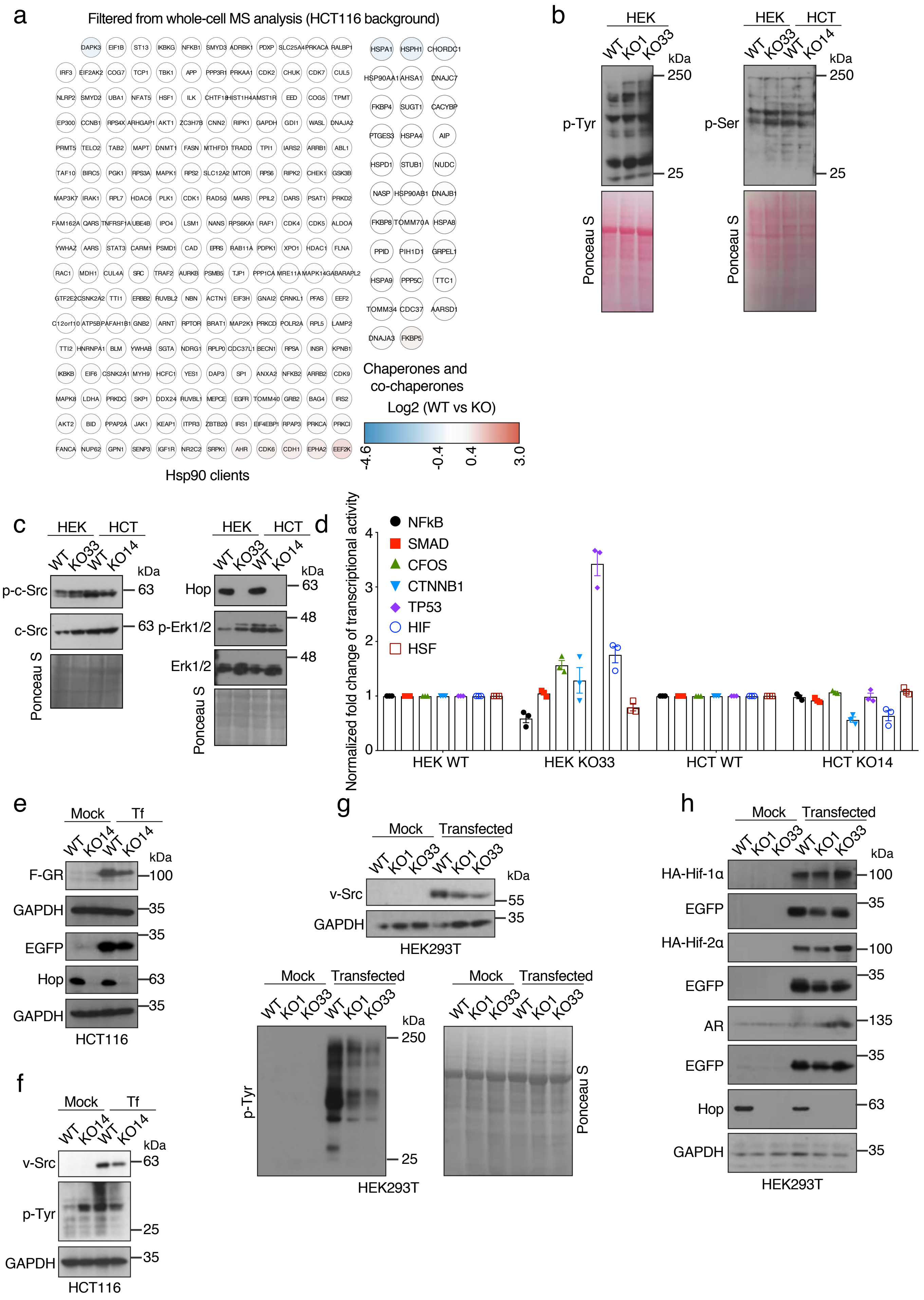

Supplementary Figure 10

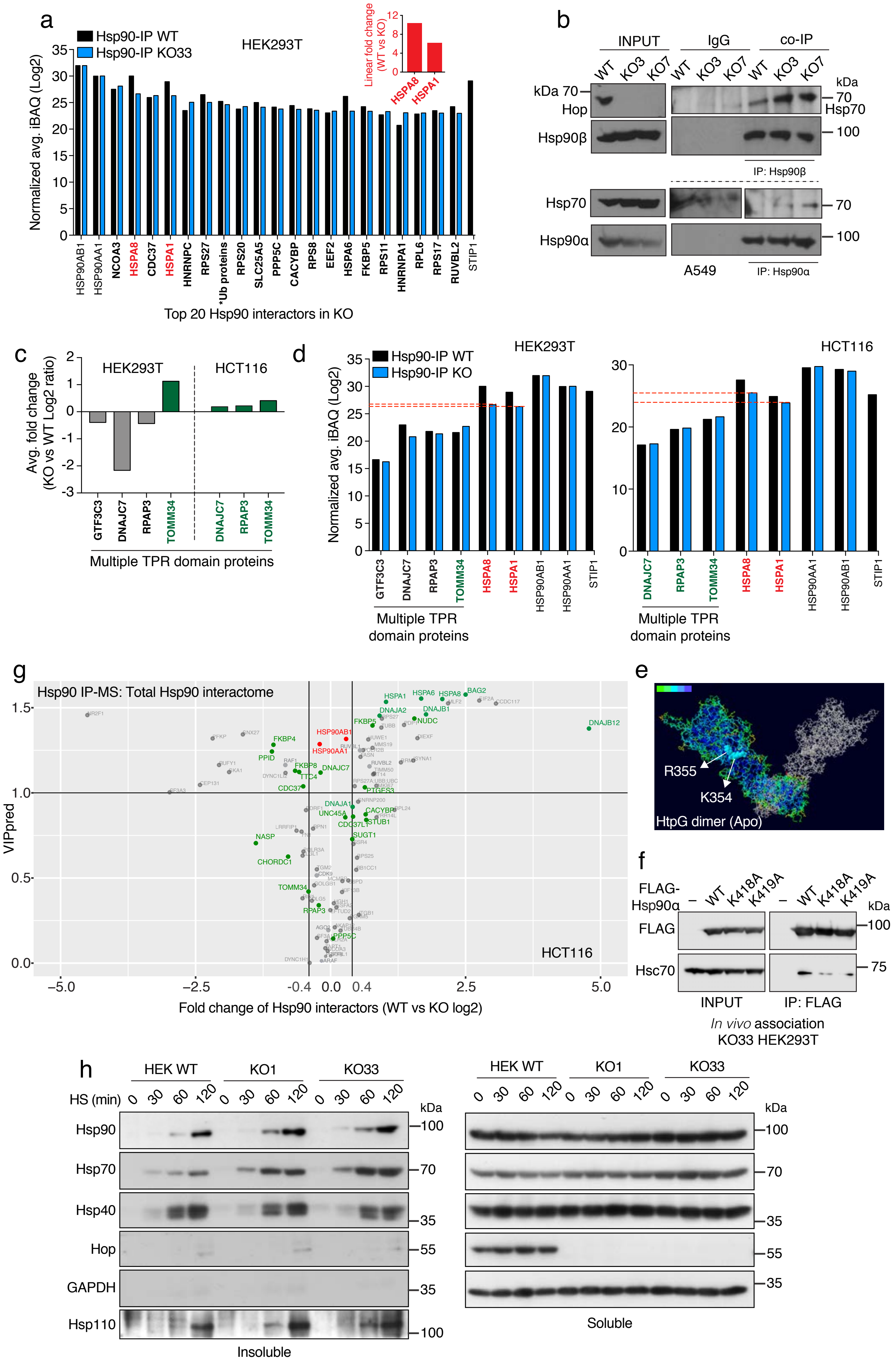

Supplementary Figure 11

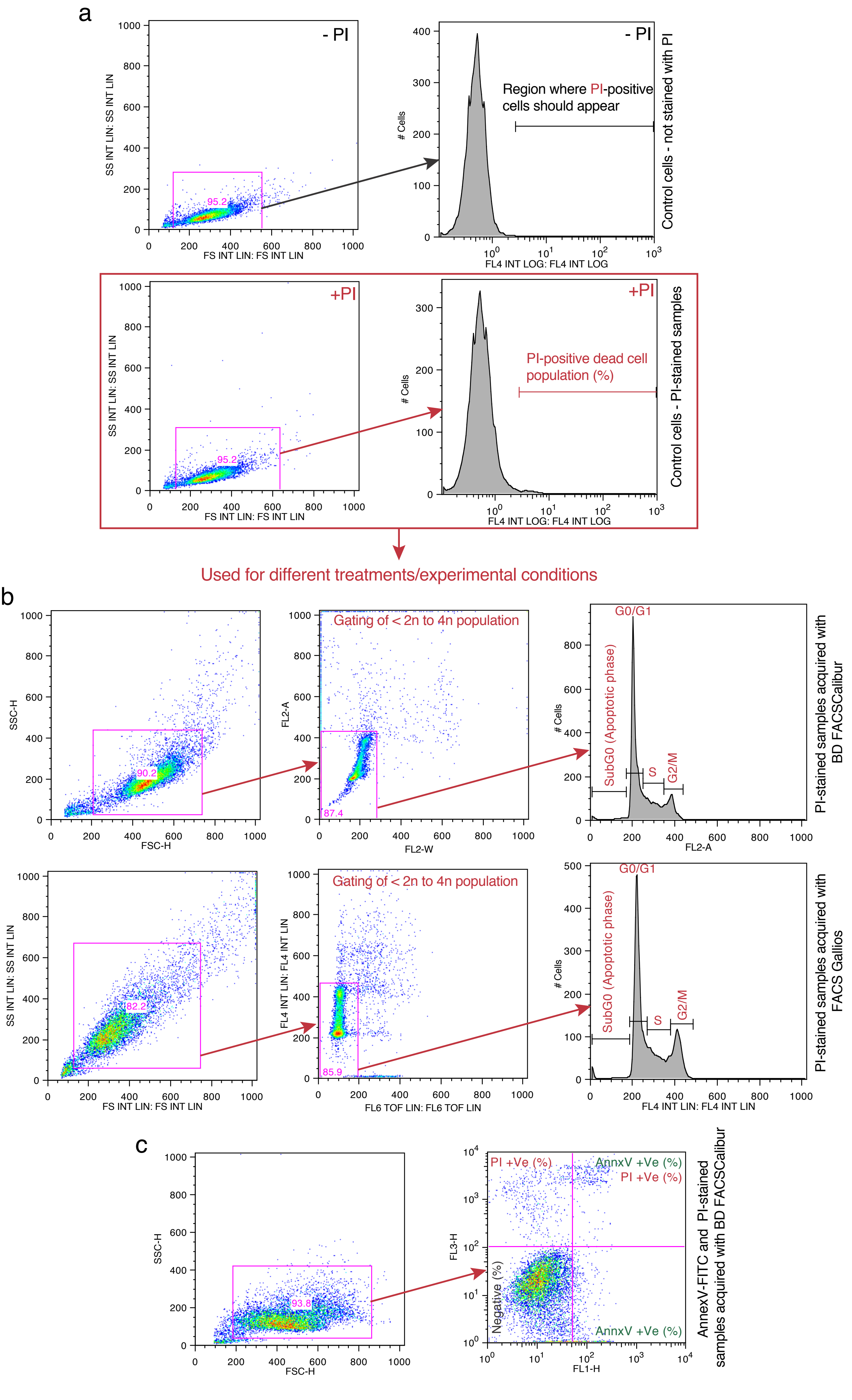

Supplementary Figure 12

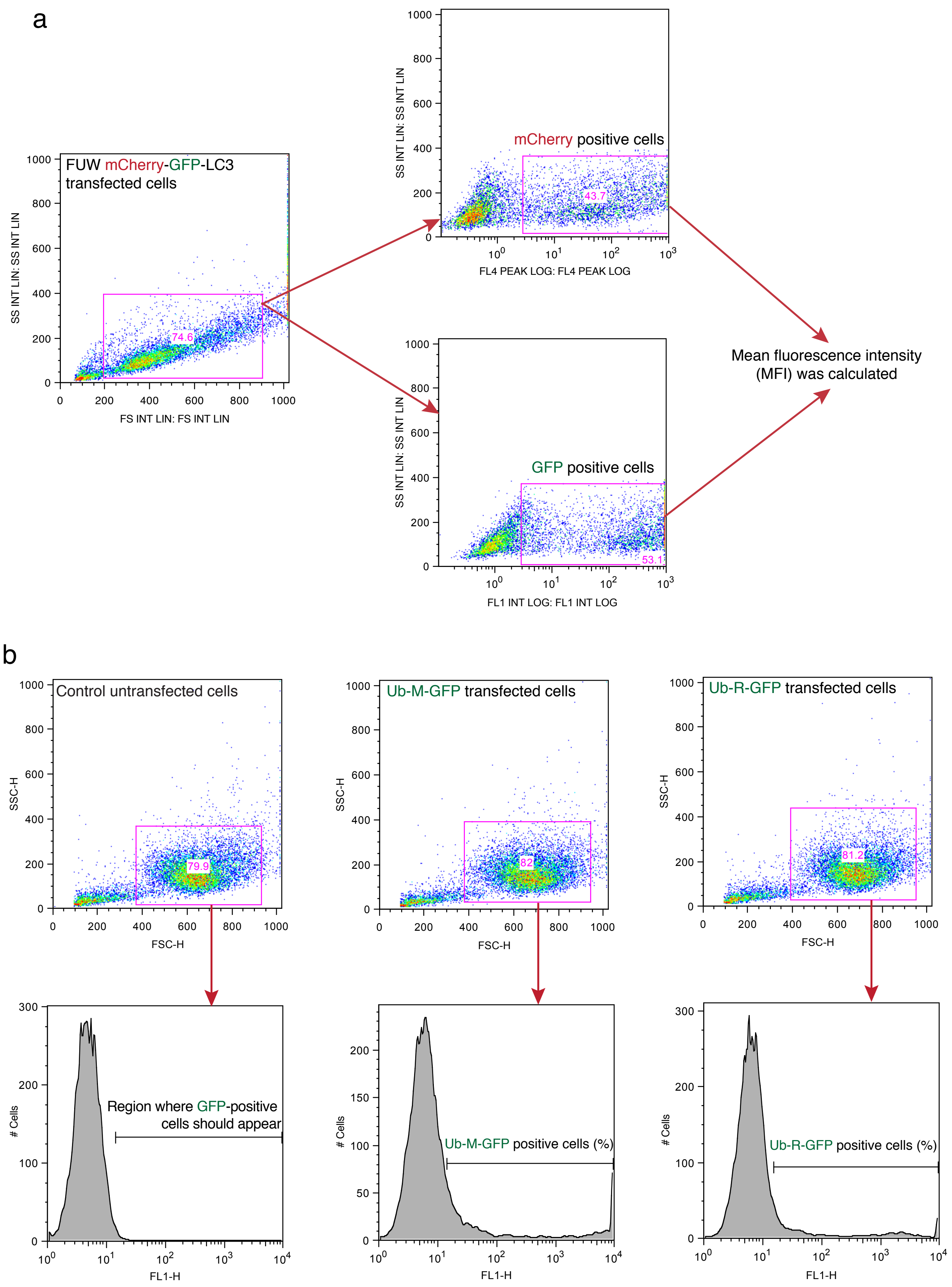

Supplement: Supplementary file 1 — Supplementary Information [file 41467_2020_19783_MOESM1_ESM.pdf]
