# Supplementary material for: The Hsp70-Hsp90 co-chaperone Hop/Stip1 shifts the proteostatic balance from folding towards degradation
Source: Nat Commun. 2020 Nov 25;11:5975. doi: 10.1038/s41467-020-19783-w (PMC7688965; doi:10.1038/s41467-020-19783-w)
Supplement: Supplementary file 8 — Reporting Summary [file 41467_2020_19783_MOESM8_ESM.pdf]

nature research

Corresponding author(s): Didier Picard

Last updated by author(s): Oct 22, 2020

Reporting Summary

Nature Research wishes to improve the reproducibility of the work that we publish. This form provides structure for consistency and transparency in reporting. For further information on Nature Research policies, see our Editorial Policies and the Editorial Policy Checklist.

Statistics

For all statistical analyses, confirm that the following items are present in the figure legend, table legend, main text, or Methods section.

n/a	Confirmed	
☐	☒	The exact sample size (*n*) for each experimental group/condition, given as a discrete number and unit of measurement
☐	☒	A statement on whether measurements were taken from distinct samples or whether the same sample was measured repeatedly
☐	☒	The statistical test(s) used AND whether they are one- or two-sided<br>*Only common tests should be described solely by name; describe more complex techniques in the Methods section.*
☒	☐	A description of all covariates tested
☒	☐	A description of any assumptions or corrections, such as tests of normality and adjustment for multiple comparisons
☐	☒	A full description of the statistical parameters including central tendency (e.g. means) or other basic estimates (e.g. regression coefficient) AND variation (e.g. standard deviation) or associated estimates of uncertainty (e.g. confidence intervals)
☐	☒	For null hypothesis testing, the test statistic (e.g. *F*, *t*, *r*) with confidence intervals, effect sizes, degrees of freedom and *P* value noted<br>*Give P values as exact values whenever suitable.*
☒	☐	For Bayesian analysis, information on the choice of priors and Markov chain Monte Carlo settings
☒	☐	For hierarchical and complex designs, identification of the appropriate level for tests and full reporting of outcomes
☒	☐	Estimates of effect sizes (e.g. Cohen's *d*, Pearson's *r*), indicating how they were calculated

Our web collection on statistics for biologists contains articles on many of the points above.

Software and code

Policy information about availability of computer code

Data collection	Mass spectrometry data acquisition was performed with Xcalibur 2.1 (Thermo Fisher).<br>FACS data were collected by using a FACSCalibur (BD) and a FACS Gallios (Beckman Coulter) flowcytometer.<br>Quantitative RT PCR data were collected by using a Bio-Rad CFX96 qPCR Instrument.
Data analysis	Cytoscape 3.7.0/3.4.0 (https://cytoscape.org/)<br>Hsp90Int.DB, Picard Lab (UNIGE https://www.picard.ch/Hsp90Int/index.php)<br>Relion 3.1<br>CellQuest Pro (BD Biosciences)<br>FlowJo 8.7 (https://www.flowjo.com/)<br>I-TASSER, Yang Zhang Lab, University of Michigan, Ann Arbor (https://zhanglab.ccmb.med.umich.edu/I-TASSER/)<br>ClusPro 2.0 (https://cluspro.org/help.php)<br>Swiss-PdbViewer 4.1.1, Swiss Institute of Bioinformatics (https://spdbv.vital-it.ch/)<br>Enricher, Ma'ayan Lab, Mount Sinai Center for Bioinformatics (http://amp.pharm.mssm.edu/Enrichr/)<br>MaxQuant 1.6.0.13, Jürgen Cox Lab, Max Planck Institute of Biochemistry (https://maxquant.org/)<br>ImageJ-Fiji<br>DinoXcope 2.0.2<br>Graph pad prism 8.0/7.0<br><br>All other details are provided in the Methods and Supplementary Methods sections.

For manuscripts utilizing custom algorithms or software that are central to the research but not yet described in published literature, software must be made available to editors and reviewers. We strongly encourage code deposition in a community repository (e.g. GitHub). See the Nature Research guidelines for submitting code & software for further information.

Data

Policy information about availability of data

All manuscripts must include a data availability statement. This statement should provide the following information, where applicable:

- Accession codes, unique identifiers, or web links for publicly available datasets
- A list of figures that have associated raw data
- A description of any restrictions on data availability

All data supporting the findings of this study are available from the corresponding authors upon reasonable request. The mass spectrometric proteomic data are available through the ProteomeXchange Consortium with the identifier PXD012774 [http://proteomecentral.proteomexchange.org/cgi/GetDataset?ID=PXD012774] and for a subset in Supplementary Data 1-4. Source data of uncropped immunoblot images and the individual data points are provided with this paper.

The UniProt human reference proteome database (of October 2017; 71,803 sequences; ftp://ftp.uniprot.org/pub/databases/uniprot/previous_releases/release-2017_10), supplemented with sequences of common contaminants, was used in the mass spectrometry experiments.

In silico protein homology modeling: The apo conformation of HtpG (PDB ID: 2IOQ) was used (https://www.rcsb.org/structure/2IOQ).

Field-specific reporting

Please select the one below that is the best fit for your research. If you are not sure, read the appropriate sections before making your selection.

☒ Life sciences ☐ Behavioural & social sciences ☐ Ecological, evolutionary & environmental sciences

For a reference copy of the document with all sections, see nature.com/documents/nr-reporting-summary-flat.pdf

Life sciences study design

All studies must disclose on these points even when the disclosure is negative.

Sample size	No sample-size calculations were performed beforehand. However, in each case, sample size was found to be adequate based on the magnitude and consistency of measurable differences between groups. For experiments where statistical significance was calculated, we chose n of at least 3.
Data exclusions	No data were excluded from analysis.
Replication	All the experiments in the article were reliably reproduced. All the attempts for the data replication were successful. Information about the number of replicates is provided in the figure legends.
Randomization	We did not perform any formal randomization techniques for in vitro experiments and this was not required for our study. Samples were distributed based on the genotypes/treatments/experimental conditions applied and their identity was known during experimentation and data analysis. No animals and/or human research participants were involved.
Blinding	Investigators were not blinded to group allocation during data collection and/or analysis. The results of the experiments provided quantitative data that were analyzed with the appropriate statistical tests to evaluate differences and statistical significance, so blinding was not relevant.

Reporting for specific materials, systems and methods

We require information from authors about some types of materials, experimental systems and methods used in many studies. Here, indicate whether each material, system or method listed is relevant to your study. If you are not sure if a list item applies to your research, read the appropriate section before selecting a response.

Materials & experimental systems

n/a	Involved in the study	
☐	☒	Antibodies
☐	☒	Eukaryotic cell lines
☒	☐	Palaeontology and archaeology
☒	☐	Animals and other organisms
☒	☐	Human research participants
☒	☐	Clinical data
☒	☐	Dual use research of concern

Methods

n/a	Involved in the study	
☒	☐	ChIP-seq
☐	☒	Flow cytometry
☒	☐	MRI-based neuroimaging

Antibodies

Antibodies used

Mouse monoclonal anti-Hop Enzo Life Sciences ADI-SRA-1500
Mouse monoclonal anti-GAPDH HyTest Ltd. 5G4
Mouse monoclonal anti-HA Biolegend 901515
Mouse monoclonal anti-FLAG Sigma F3165
Mouse monoclonal anti-His Sigma H1029
Mouse monoclonal anti-Hsp70 StressMarq SMC-100
Mouse monoclonal anti-Hsc70 StressMarq SMC-151
Rabbit polyclonal anti-Raf1 Santa Cruz Biotechnology Sc-133
Rabbit polyclonal anti-Psmd1 Bethyl A303-851A-T
Rabbit polyclonal anti-Psmd2 Bethyl A303-854A-T
Rabbit polyclonal anti-Psmd6 Enzo Lifesciences BML-PW8225
Rabbit polyclonal anti-Psmd9 Bethyl A304-979A-T
Rabbit polyclonal anti-Psmd5 Bethyl A304-999A-T
Rabbit polyclonal anti-Paaf1 Abcam Ab103566
Mouse monoclonal anti-Psmc5 Enzo Lifesciences BML-PW9265
Mouse monoclonal anti-alpha-Tubulin Merck Millipore CP06
Mouse monoclonal anti-Psma3 Enzo Lifesciences BML-PW8110
Rabbit polyclonal anti 20S CP Enzo Lifesciences BML-PW8155
Mouse monoclonal anti-Ub ThermoFisher Sci. 13-1600
Mouse monoclonal anti-EGFP Roche 11814460001
Mouse monoclonal anti-p-Tyr Cell Signaling Technology 9411
Mouse monoclonal anti-p-Ser Santa Cruz Biotechnology Sc-81514
Rat monoclonal anti-Hsp90Alpha (9D2) Enzo Lifesciences ADI-SPA-840
Mouse monoclonal anti-Hsp90Beta (H90-10) Toft Lab, Mayo clinic/Gift, StressMarq/SMC-107
Mouse monoclonal anti-p23 (JJ3) Toft Lab, Mayo Clinic/Gift, ThermoFisher/MA3-414
Rabbit monoclonal anti-Cdc37 Cell Signaling Technology 4793
Rabbit polyclonal anti-Akt Cell Signaling Technology 9272
Mouse monoclonal anti-Cdk2 Santa Cruz Biotechnology Sc-53220
Rabbit polyclonal anti-Erbb4 Santa Cruz Biotechnology Sc-283
Rabbit polyclonal anti-Hsf1 Enzo Lifesciences ADI-SPA-901
Mouse monoclonal anti-p53 Santa Cruz Biotechnology Sc-126
Rabbit polyclonal anti-Beta-catenin Sigma C2206
Rabbit polyclonal anti-Stat3 Cellomics - (Discontinued)
Rabbit polyclonal Anti-BclXl Santa Cruz Biotechnology Sc-634
Rabbit polyclonal Anti-Bcl2 Santa Cruz Biotechnology Sc-783
Rabbit polyclonal anti-HDAC6 Santa Cruz Biotechnology Sc-11420
Mouse monoclonal anti-vimentin Abcam Ab8069
Mouse monoclonal anti-v-Src EMD Millipore 05-185
Rabbit polyclonal anti-progesterone receptor Bethyl A301-200A-T
Rabbit polyclonal anti-glucocorticoid receptor Santa Cruz Biotechnology Sc-8992
Rabbit polyclonal anti-androgen receptor Bethyl A303-965A-T
Mouse polyclonal anti-Sti1 (Yeast) Toft Lab, Mayo Clinic Gift
Mouse monoclonal anti-Cdk4 NeoMarkers MS-616-P1
Rabbit monoclonal anti-p-c-Src (Y416) Cell Signaling Technology 6943
Rabbit polyclonal anti-c-Src Cell Signaling Technology 2108
Rabbit polyclonal anti-H2A.Z Abcam Ab4174
Mouse monoclonal anti-p-Erk Santa Cruz Biotechnology Sc-7383
Rabbit polyclonal anti-Erk2 Santa Cruz Biotechnology Sc-154
Rabbit polyclonal anti-Hsp40/Hdj1 Enzo Lifesciences ADI-SPA-400
Rabbit polyclonal anti-Hsp110 Enzo Lifesciences ADI-SPA-1101
Mouse monoclonal anti-Puromycin Sigma/Millipore MABE343
Mouse polyclonal anti-Rpt1 Abcam Ab21743
Rabbit polyclonal anti-Rpt2 Abcam Ab22679
Rabbit polyclonal anti-Hsp90a Thermo Fisher Sci. PA3-013
Anti-rat IgG-HRP Invitrogen 629520
Anti-mouse IgG-HRP Invitrogen 31430
Anti-rabbit IgG-HRP Invitrogen 31460
Anti-mouse IgM-HRP Enzo Lifesciences ADI-SAB-110-J
Normal rat IgG Santa Cruz Biotechnology Sc-2026
Normal mouse IgG Sigma/Millipore I5381/12-371
Normal rabbit IgG Sigma I5006
Anti-mouse IgG-Alexa Fluor 488 Invitrogen A11017
Anti-rabbit IgG-Alexa Fluor 488 Invitrogen A11034

Validation

Mouse monoclonal anti-Hop Enzo Life Sciences ADI-SRA-1500: Knockout validated in this paper. Detects human antigen, western blot (WB) and immunoprecipitation (IP) compatible.
Mouse monoclonal anti-GAPDH HyTest Ltd. 5G4: Hybridoma clones have been derived from hybridization of Sp2/0 myeloma cells with spleen cells of Balb/c mice immunized with human or rabbit GAPDH. Detects human antigen, WB compatible.
Mouse monoclonal anti-HA Biolegend 901515: Validated by Western blot analysis of cell lysates from CHO and CHO-HA stable cells using HA Mouse primary antibody. Also validated in this paper. WB and IP compatible.
Mouse monoclonal anti-FLAG Sigma F3165: Detects specifically FLAG-tagged recombinant protein expressed in the cells which is also showed in this paper. WB and IP compatible.

Mouse monoclonal anti-His Sigma H1029: The antibody recognizes synthetic poly-Histidine, as well as native or denatured, reduced forms of proteins tagged with 6X histidines, expressed in selected vectors. WB compatible.
Mouse monoclonal anti-Hsp70 StressMarq SMC-100: Validation has been done by WB using several human cell lysates. Detects human antigen, WB compatible.
Mouse monoclonal anti-Hsc70 StressMarq SMC-151: Validation has been done by WB using several human cell lysates and the purified protein. Detects human antigen, WB compatible.
Rabbit polyclonal anti-Raf1 Santa Cruz Biotechnology Sc-133: Epitope mapping at the C-terminus of Raf-1 of human origin. Köhler, M. et al. 2016. The EMBO journal. 35: 143-61. Discontinued.
Rabbit polyclonal anti-Psmd1 Bethyl A303-851A-T: Validation has been done by WB using several human cell lysates and also by IP. Detects human antigen, WB compatible.
Rabbit polyclonal anti-Psmd2 Bethyl A303-854A-T: Validation has been done by WB using several human cell lysates and also by IP. Detects human antigen, WB compatible.
Rabbit polyclonal anti-Psmd6 Enzo Lifesciences BML-PW8225: This antibody is used for WB in the paper J. Proteomics 139, 45 (2016). Detects human and yeast antigen, WB compatible.
Rabbit polyclonal anti-Psmd9 Bethyl A304-979A-T: Validation has been done by WB using several human cell lysates. Detects human antigen, WB compatible.
Rabbit polyclonal anti-Psmd5 Bethyl A304-999A-T: Validation has been done by WB using several human cell lysates and also by IP. Detects human antigen, WB compatible.
Rabbit polyclonal anti-Paaf1 Abcam Ab103566: Synthetic peptide conjugated to KLH, between N-terminal amino acids 115-145 of Human PAAF1 was used as immunogen. Validation has been done by WB using human cell lysates. Detects human antigen, WB compatible.
Mouse monoclonal anti-Psmc5 Enzo Lifesciences BML-PW9265: Immunogen is recombinant human Psmc5 protein. Validation has been done by WB using human cell lysates. Detects human antigen, WB and IHC compatible.
Mouse monoclonal anti-alpha-Tubulin Merck Millipore CP06: This Anti-α-Tubulin Mouse mAb (DM1A) is validated for use in Immunoblotting, Immunofluorescence for the detection of α-Tubulin. Detects human antigen, WB compatible.
Mouse monoclonal anti-Psma3 Enzo Lifesciences BML-PW8110: Recognizes the α7 subunit of the 20S proteasome. Detects human and yeast antigen, WB and IHC compatible.
Rabbit polyclonal anti 20S CP Enzo Lifesciences BML-PW8155: Validated with human erythrocyte-derived 20S proteasome lysate by WB. Detects human and yeast antigen, WB and IHC compatible.
Mouse monoclonal anti-Ub ThermoFisher Sci. 13-1600: Validated with lysates from cells treated with MG132 proteasomal inhibitor by WB. Detects human antigen, WB compatible.
Mouse monoclonal anti-EGFP Roche 11814460001: Anti-GFP is tested for functionality and purity relative to a reference standard to confirm the quality of each new reagent preparation. WB compatible.
Mouse monoclonal anti-p-Tyr Cell Signaling Technology 9411: Phospho-Tyrosine Mouse mAb (P-Tyr-100) is a high affinity antibody. ELISAs against a wide variety of phosphopeptides indicate that P-Tyr-100 binds phospho-Tyr in a manner largely independent of the surrounding amino acid sequence. WB compatible.
Mouse monoclonal anti-p-Ser Santa Cruz Biotechnology Sc-81514: p-Ser Antibody (16B4) is a high quality monoclonal p-Ser antibody (also designated phosphorylated serine antibody, phospho serine antibody, or p-serine antibody) suitable for the detection of the p-Ser protein of mouse, rat, human and canine origin.
Rat monoclonal anti-Hsp90Alpha (9D2) Enzo Lifesciences ADI-SPA-840: This antibody was validated by immunoblot analysis using purified human Hsp90alpha recombinant protein along with human Hsp90beta recombinant protein as a negative control. Knockout validated in this paper. Detects human antigen, WB and IP compatible.
Mouse monoclonal anti-Hsp90Beta (H90-10) Toft Lab, Mayo clinic/Gift, StressMarq/SMC-107: See below
Mouse monoclonal anti-p23 (JJ3) Toft Lab, Mayo Clinic/Gift, ThermoFisher/MA3-414: See below
Rabbit monoclonal anti-Cdc37 Cell Signaling Technology 4793: Validation has been done by WB using several human cell lysates. Detects human antigen, WB compatible.
Rabbit polyclonal anti-Akt Cell Signaling Technology 9272: Knockdown validated. Detects human antigen, WB compatible.
Mouse monoclonal anti-Cdk2 Santa Cruz Biotechnology Sc-53220: Raised against human recombinant Cdk2. Validation has been done by WB using several human cell lysates. Detects human antigen, WB compatible.
Rabbit polyclonal anti-Erbb4 Santa Cruz Biotechnology Sc-283: Validation has been done by WB using Erbb4 overexpressed cell lysates. Detects human antigen, WB compatible.
Rabbit polyclonal anti-Hsf1 Enzo Lifesciences ADI-SPA-901: Recombinant human HSF1 was used as immunogen. Validation has been done by WB using heat shocked and non-heat shocked cell lysates. Detects human antigen, WB compatible.
Mouse monoclonal anti-p53 Santa Cruz Biotechnology Sc-126: Validation has been done by WB using several human cell lysates. Detects human antigen, WB compatible.
Rabbit polyclonal anti-Beta-catenin Sigma C2206: Knockdown validated. Detects human antigen, WB compatible.
Rabbit polyclonal anti-Stat3 Cellomics - (Discontinued)
Rabbit polyclonal Anti-BclXl Santa Cruz Biotechnology Sc-634: Epitope mapping at the N-terminus of Bcl-xS/L of human origin. Masotti, A. et al. 2015. BMC genomics. 16: 480. Discontinued.
Rabbit polyclonal Anti-Bcl2 Santa Cruz Biotechnology Sc-783: Aguayo-Mazzucato, C. et al. 2018. Diabetes. 67: 1322-1331. Discontinued.
Rabbit polyclonal anti-HDAC6 Santa Cruz Biotechnology Sc-11420: Epitope corresponding to amino acids 916-1215 mapping at the C-terminus of HDAC6 of human origin. Ehnert, S. et al. 2017. Journal of molecular medicine (Berlin, Germany). 95: 653-663. Discontinued.
Mouse monoclonal anti-vimentin Abcam Ab8069: Knockout validated. Detects human antigen, WB compatible.
Mouse monoclonal anti-v-Src EMD Millipore 05-185: Routinely evaluated in (60kDa) on a RIPA lysate of chicken embryo fibroblasts (CEF). This antibody is specific only for avian Src; this antibody is excellent for distinguishing pp60src in cells transfected with v-src or c-src expression vectors. WB compatible.
Rabbit polyclonal anti-progesterone receptor Bethyl A301-200A-T: Validation has been done by WB using several human cell lysates and also by IP. Detects human antigen, WB compatible.
Rabbit polyclonal anti-glucocorticoid receptor Santa Cruz Biotechnology Sc-8992: Epitope corresponding to amino acids 121-420 mapping within an internal region of GR of human origin. Chen, H. et al. 2017. Molecular brain. 10: 12. Discontinued.
Rabbit polyclonal anti-androgen receptor Bethyl A303-965A-T: Validation has been done by WB using several human cell lysates and also by IP. Detects human antigen, WB compatible.
Mouse polyclonal anti-Sti1 (Yeast) Toft Lab, Mayo Clinic Gift: See below
Mouse monoclonal anti-Cdk4 NeoMarkers MS-616-P1: Immunogen was purified recombinant cdk4 protein. Validation has been done

by WB using several human cell lysates. Detects human antigen, WB compatible.
Rabbit monoclonal anti-p-c-Src (Y416) Cell Signaling Technology 6943: Validation has been done by WB using growth factor treated and untreated cell lysates. Detects human antigen, WB compatible.
Rabbit polyclonal anti-c-Src Cell Signaling Technology 2108: Validation has been done by WB using several human cell lysates. Detects human antigen, WB compatible.
Rabbit polyclonal anti-H2A.Z Abcam Ab4174: Validation has been done by WB using several human cell lysates and nuclear extracts. Detects human antigen, WB compatible.
Mouse monoclonal anti-p-Erk Santa Cruz Biotechnology Sc-7383: Validation has been done by WB using UV-irradiated cell lysates as a positive control. Detects human antigen, WB compatible.
Rabbit polyclonal anti-Erk2 Santa Cruz Biotechnology Sc-154: Epitope mapping at the C-terminus of ERK2 of rat origin. Tam, K.W. et al. 2018. Oncotarget. 9: 4593-4606. Diacontinued.
Rabbit polyclonal anti-Hsp40/Hdj1 Enzo Lifesciences ADI-SPA-400: Validation has been done by WB using heat shocked and non-heat shocked cell lysates and also with purified recombinant protein. Detects human antigen, WB compatible.
Rabbit polyclonal anti-Hsp110 Enzo Lifesciences ADI-SPA-1101: Synthetic peptide corresponding to a portion of hamster HSP110 was used as immunogen. The sequence is completely conserved in human and mouse. Detects human antigen, WB compatible.
Mouse monoclonal anti-Puromycin Sigma/Millipore MABE343: Anti-Puromycin antibody detects puromycin incorporated protein and can be inhibited by cycloheximide. WB compatible.
Mouse polyclonal anti-Rpt1 Abcam Ab21743: Validation has been done by WB using Rpt1 expressed E. coli extracts. Detects yeast antigen, WB compatible.
Rabbit polyclonal anti-Rpt2 Abcam Ab22679: Validation has been done by WB using purified yeast 26S proteasome preparation. Detects yeast antigen, WB compatible.
Rabbit polyclonal anti-Hsp90a Thermo Fisher Sci. PA3-013: Validation has been done by WB using several human cell lysates. Detects human antigen, WB compatible. In our experience this antibody is not specific to Hsp90alpha anymore. We used this antibody to detect total Hsp90.

We validated the antibodies received as gifts by ourselves. Anti-mouse Hsp90beta H90-10 antibody was validated by immunoblot analysis using purified human Hsp90beta recombinant protein along with human Hsp90alpha recombinant protein as a negative control. Also this antibody is knockout validated in this paper.
The mouse anti-Sti1 (Yeast) antibody was validated by immunoblot analysis using WT and STI1-KO yeast cell extracts. The data are presented in the paper. This antibody is not commercially available. This could be obtained from the corresponding author upon a request.
The mouse anti-p23 antibody was validated by immunoblot analysis using several human cell extracts.

Eukaryotic cell lines

Policy information about cell lines

Cell line source(s)	HEK293T, HCT116, and A549 cell lines were purchased from the American Type Culture Collection (ATCC). The yeast strains BY4741, Y01803, and BMA64-1A were purchased from Euroscarf.
Authentication	Cell lines were not authenticated by ourselves.
Mycoplasma contamination	All cell lines were tested to be mycoplasma negative.
Commonly misidentified lines (See ICLAC register)	None of the cell lines used in this study were found in the commonly misidentified cell lines database.

Flow Cytometry

Plots

Confirm that:

☒ The axis labels state the marker and fluorochrome used (e.g. CD4-FITC).

☒ The axis scales are clearly visible. Include numbers along axes only for bottom left plot of group (a 'group' is an analysis of identical markers).

☒ All plots are contour plots with outliers or pseudocolor plots.

☒ A numerical value for number of cells or percentage (with statistics) is provided.

Methodology

Sample preparation	Described in detail in Methods section for each experiment.
Instrument	FACSCalibur from Becton Dickinson. FACS Gallios from Beckman Coulter.
Software	Data acquisition was done by CellQuestPro software in FACSCalibur. All data analyses were performed with FlowJo.
Cell population abundance	We only used the flow cytometry to analyze the cells, not to sort them. For all flow cytometric experiments we acquired/ analyzed at least 10,000 cells per sample.
Gating strategy	Preliminary cell population was gated from FSC/SSC plot for all the flow cytometry experiments. Boundaries of the positive

Gating strategy

and negative populations were determined by using unstained cells. For the cell cycle analyses, different cell cycle phases were determined by the population density and population distribution.

☒ Tick this box to confirm that a figure exemplifying the gating strategy is provided in the Supplementary Information.
