# Supplementary material for: The Hsp70-Hsp90 co-chaperone Hop/Stip1 shifts the proteostatic balance from folding towards degradation
Source: Nat Commun. 2020 Nov 25;11:5975. doi: 10.1038/s41467-020-19783-w (PMC7688965; doi:10.1038/s41467-020-19783-w)

Figure 1

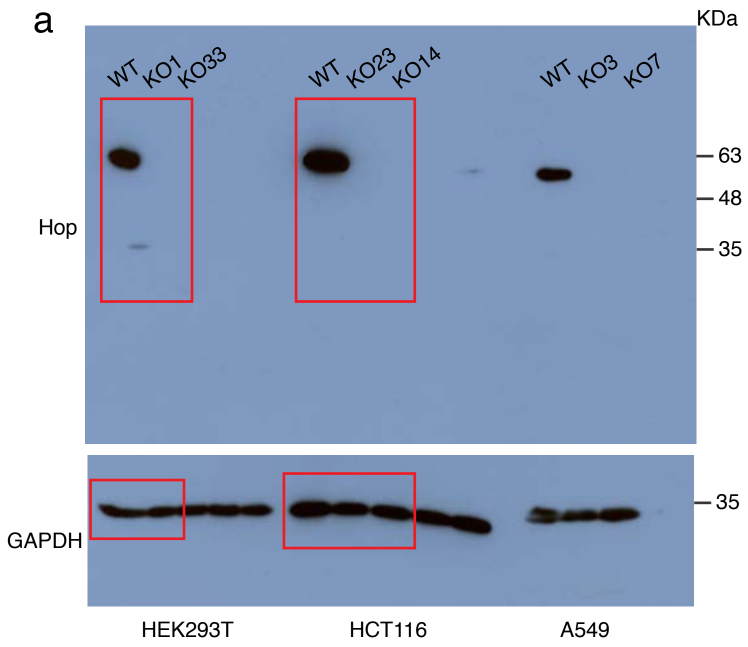
a
KDa
WT
KO1
KO33
WT
KO23
KO14
WT
KO3
KO7
63
48
35
Hop
35
GAPDH
HEK293T
HCT116
A549

Figure 3

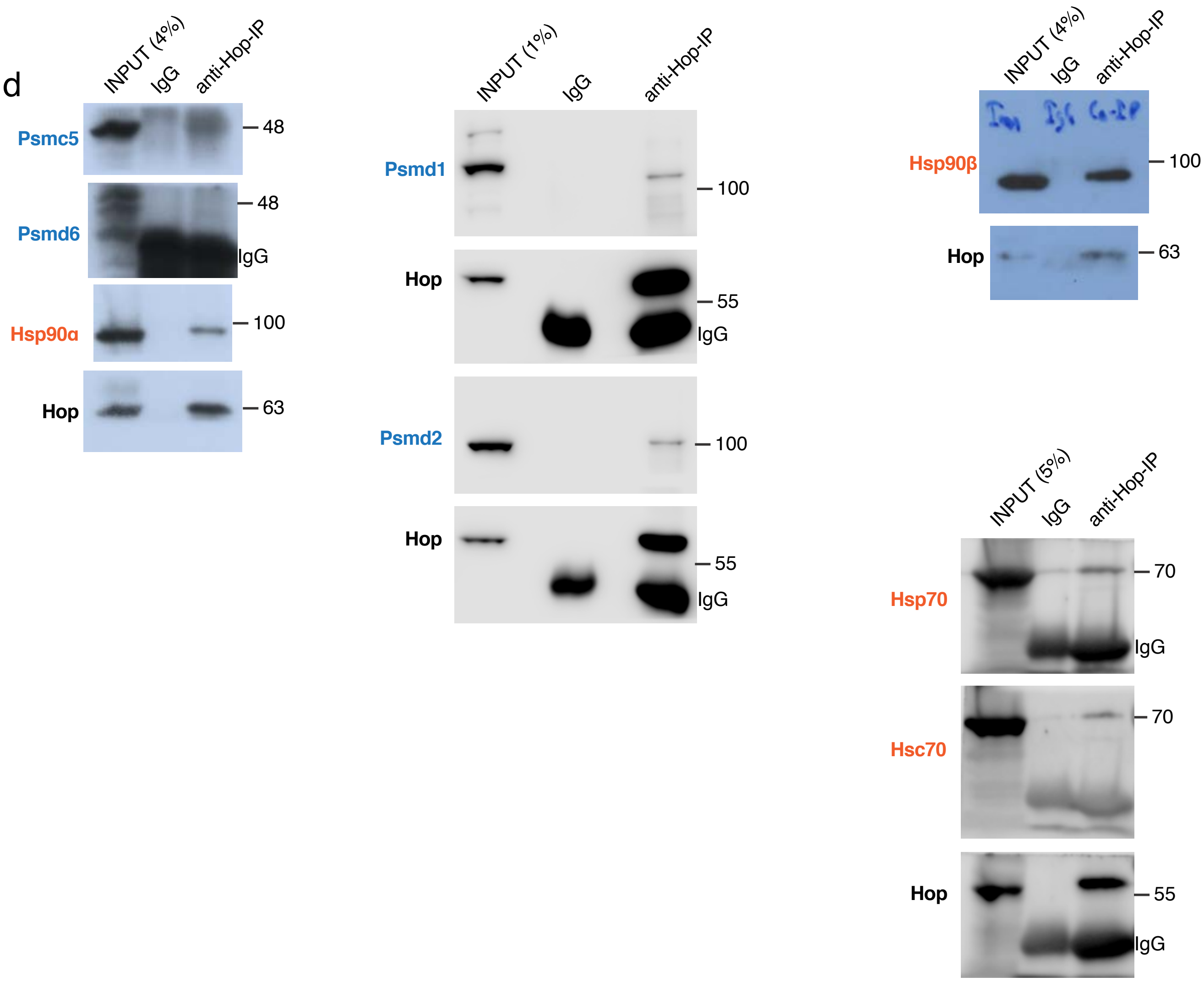
d
INPUT (4%)
IgG
anti-Hop-IP
Psmc5
48
Psmd6
48
IgG
Hsp90α
100
Hop
63
INPUT (1%)
IgG
anti-Hop-IP
Psmd1
100
Hop
55
IgG
Psmd2
100
Hop
55
IgG
INPUT (4%)
IgG
anti-Hop-IP
Hsp90β
100
Hop
63
INPUT (5%)
IgG
anti-Hop-IP
Hsp70
70
IgG
Hsc70
70
Hop
55
IgG

Figure 4

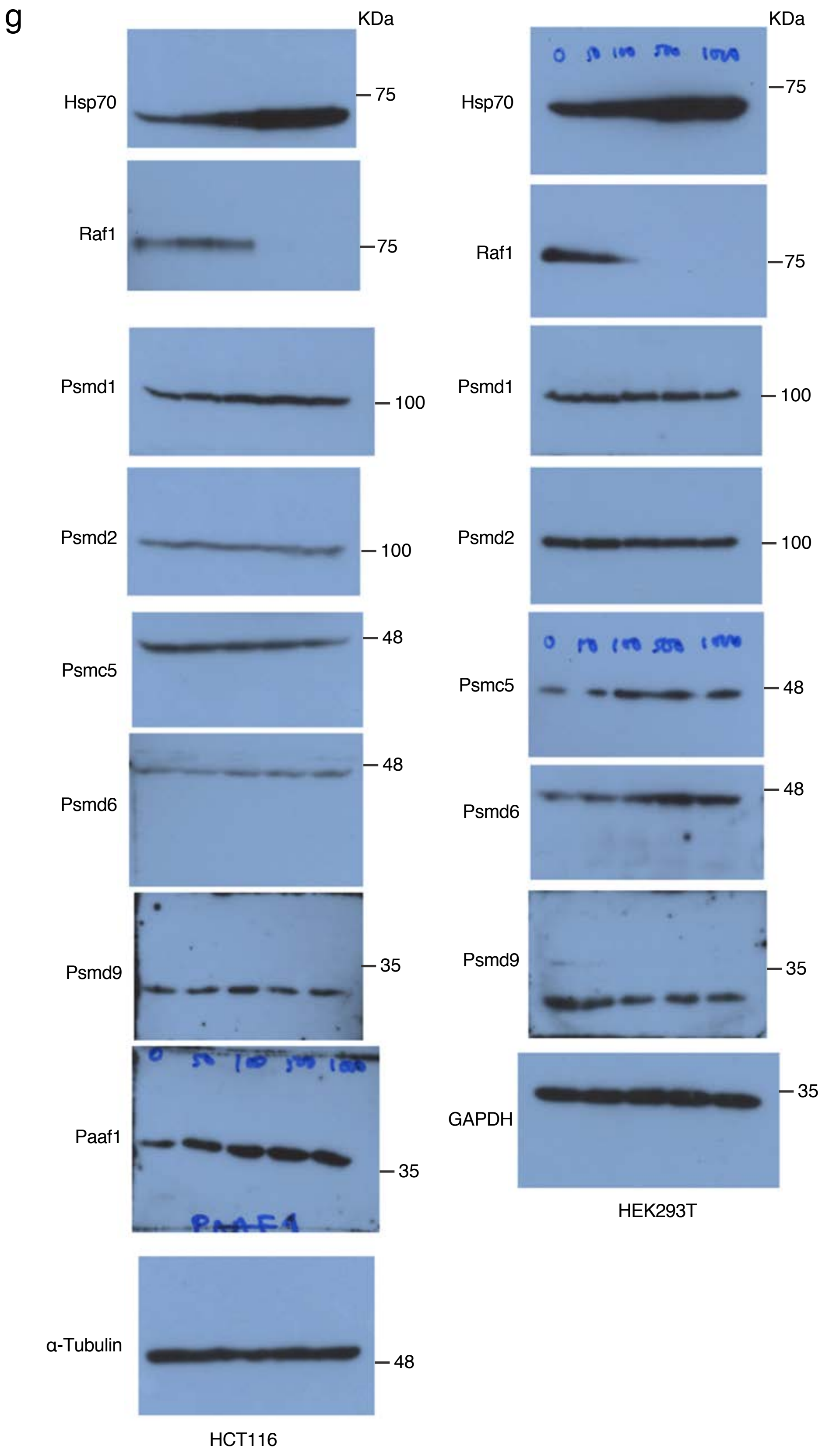
g
KDa
Hsp70
75
Raf1
75
Psmd1
100
Psmd2
100
Psmc5
48
Psmd6
48
Psmd9
35
0 50 100 500 1000
Paaf1
35
PAAF1
α-Tubulin
48
HCT116
KDa
0 50 100 500 1000
Hsp70
75
Raf1
75
Psmd1
100
Psmd2
100
0 50 100 500 1000
Psmc5
48
Psmd6
48
Psmd9
35
GAPDH
35
HEK293T

Figure 5

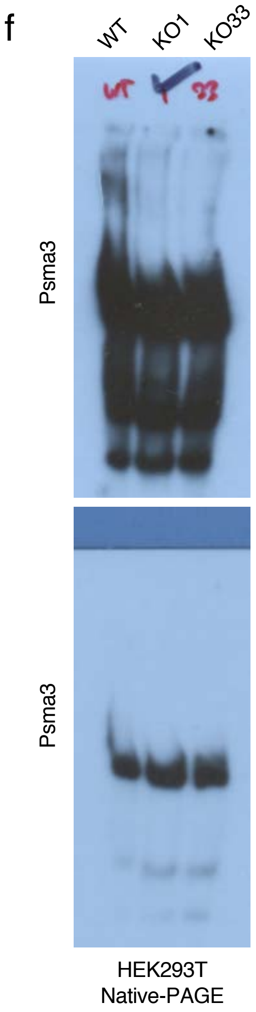
f
WT
KO1
KO33
Psma3
Psma3
HEK293T
Native-PAGE

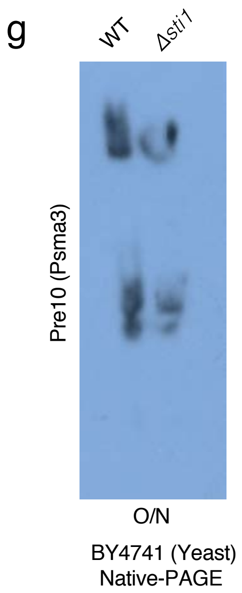
g
WT
Δsti1
Pre10 (Psma3)
O/N
BY4741 (Yeast)
Native-PAGE

Figure 6

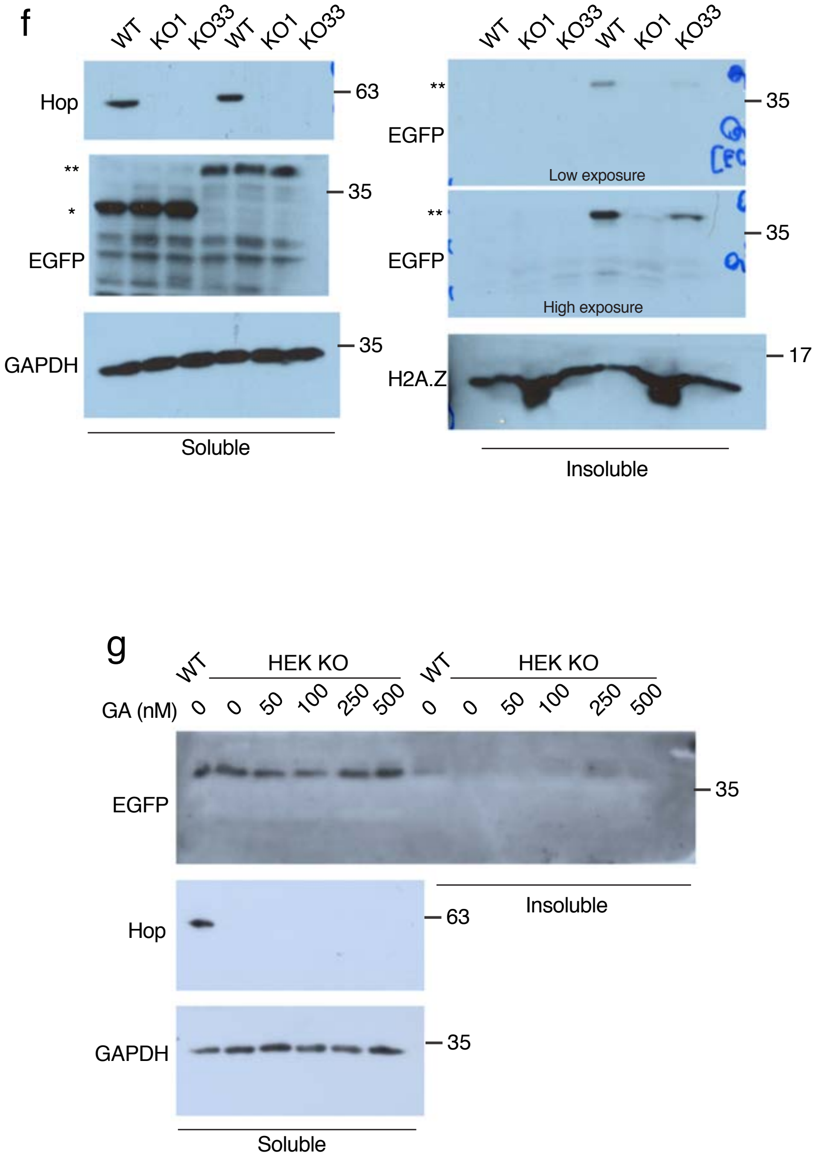
f
WT
KO1
KO33
WT
KO1
KO33
Hop
63
**
*
EGFP
35
GAPDH
35
Soluble
WT
KO1
KO33
WT
KO1
KO33
**
EGFP
35
Low exposure
**
EGFP
35
High exposure
H2A.Z
17
Insoluble
g
WT
HEK KO
WT
HEK KO
GA (nM)
0
0
50
100
250
500
0
0
50
100
250
500
EGFP
35
Insoluble
Hop
63
GAPDH
35
Soluble

Figure 7

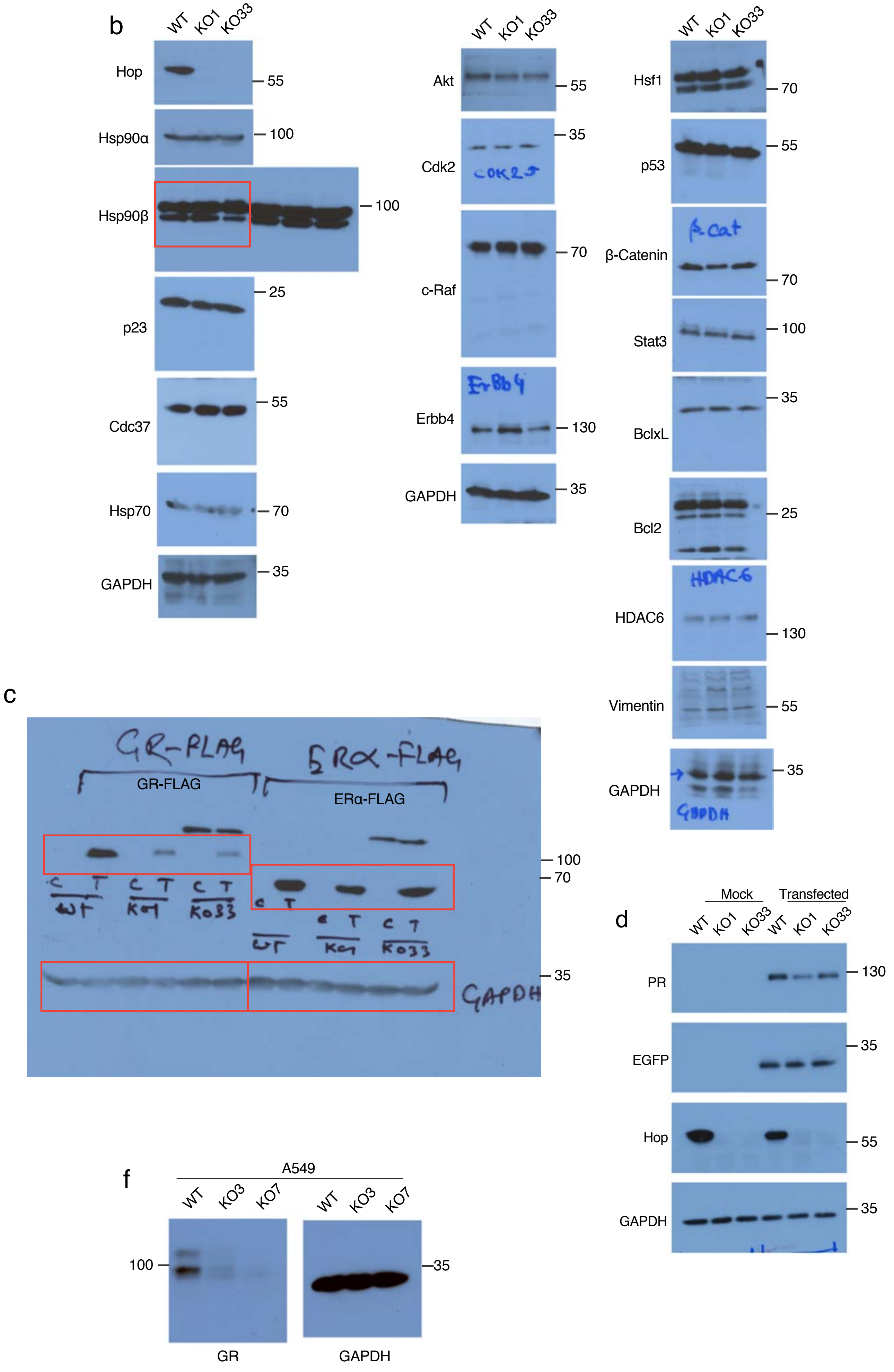
b
WT KO1 KO33
Hop
55
Hsp90α
100
Hsp90β
100
p23
25
Cdc37
55
Hsp70
70
GAPDH
35
Akt
55
Cdk2
35
c-Raf
70
Erbb4
130
GAPDH
35
Hsf1
70
p53
55
β-Catenin
70
Stat3
100
BclxL
35
Bcl2
25
HDAC6
130
Vimentin
55
GAPDH
35
c
GR-FLAG
ERα-FLAG
100
70
35
d
Mock
Transfected
WT KO1 KO33 WT KO1 KO33
PR
130
EGFP
35
Hop
55
GAPDH
35
f
A549
WT KO3 KO7 WT KO3 KO7
100
35
GR
GAPDH

Figure 8

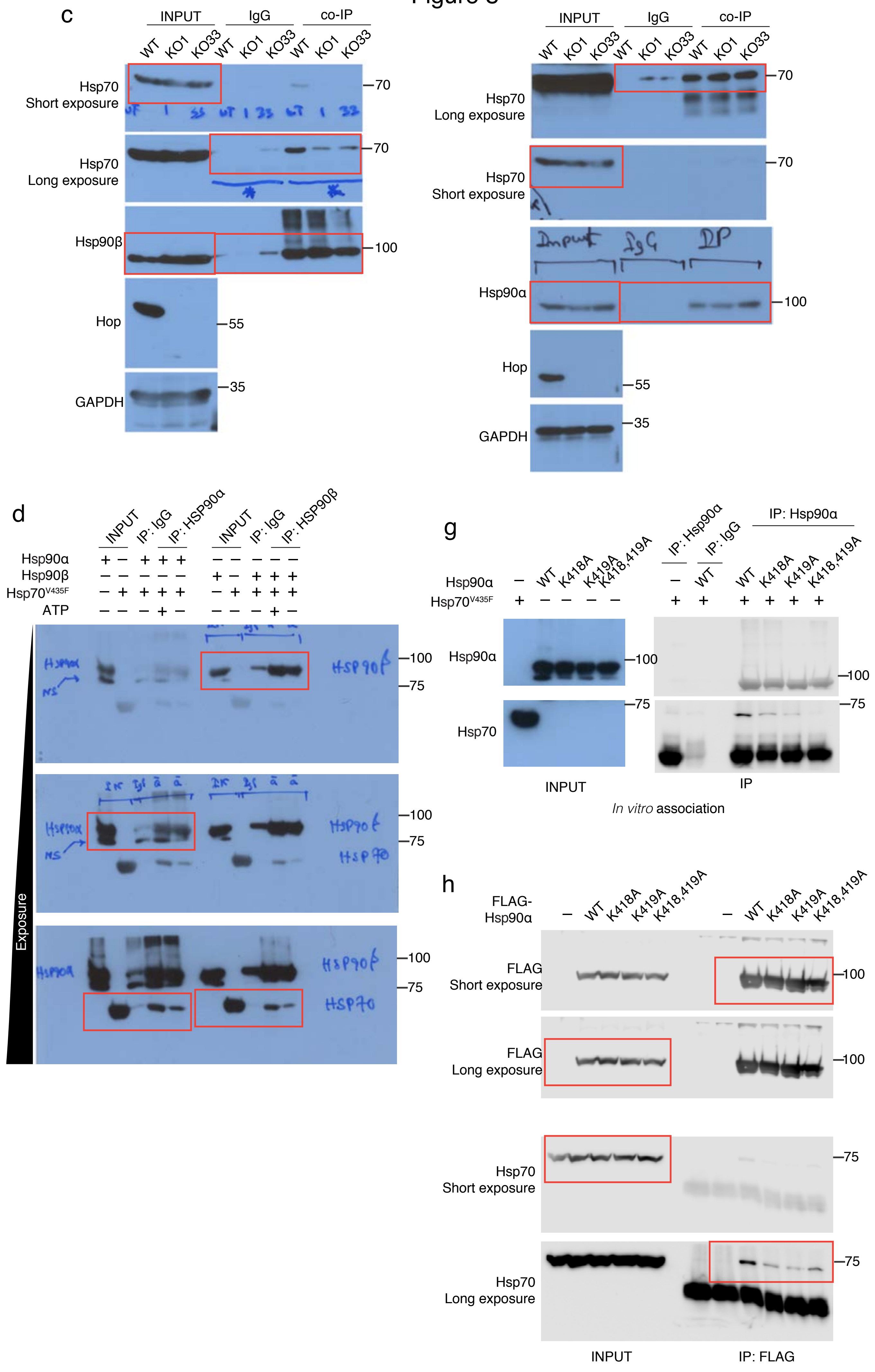
c
INPUT
IgG
co-IP
WT KO1 KO33 WT KO1 KO33 WT KO1 KO33
Hsp70 Short exposure
Hsp70 Long exposure
Hsp90β
Hop
GAPDH
70
100
55
35
Hsp70 Long exposure
Hsp70 Short exposure
Hsp90α
d
INPUT IP: IgG IP: HSP90α INPUT IP: IgG IP: HSP90β
Hsp90α
Hsp90β
Hsp70V435F
ATP
Exposure
100
75
g
IP: Hsp90α
IP: IgG
WT K418A K419A K418,419A
Hsp90α
Hsp70V435F
Hsp70
INPUT
IP
In vitro association
h
FLAG-Hsp90α
FLAG Short exposure
FLAG Long exposure
Hsp70 Short exposure
Hsp70 Long exposure
IP: FLAG

Supplementary figure 2

i

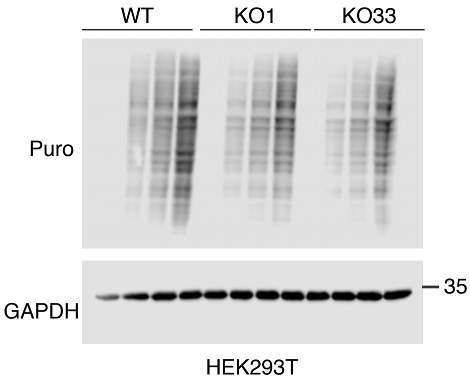

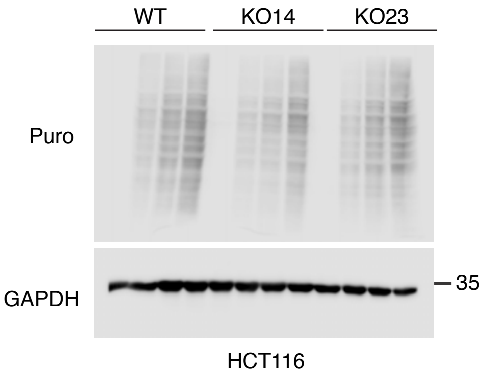

Supplementary figure 3

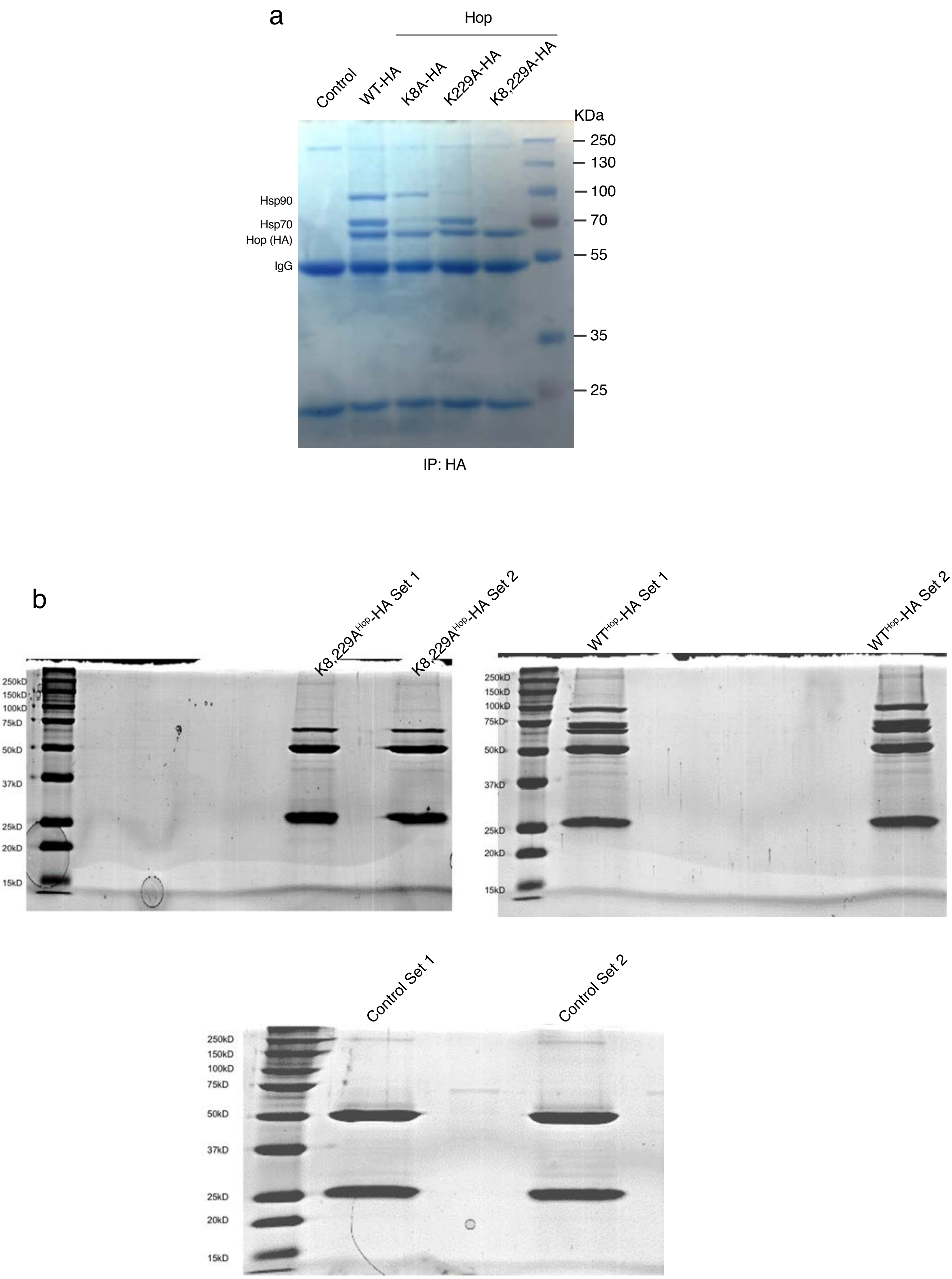

Supplementary figure 4

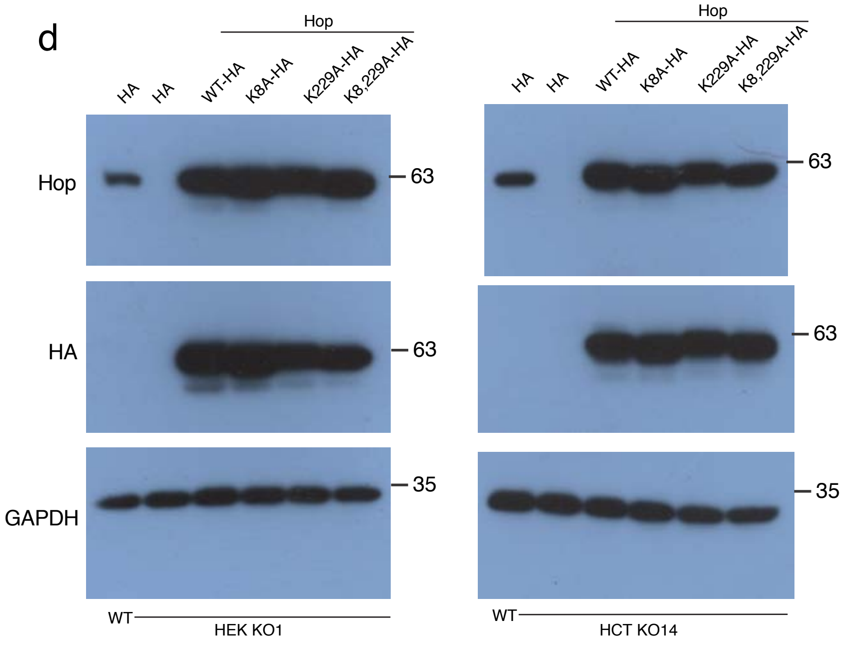

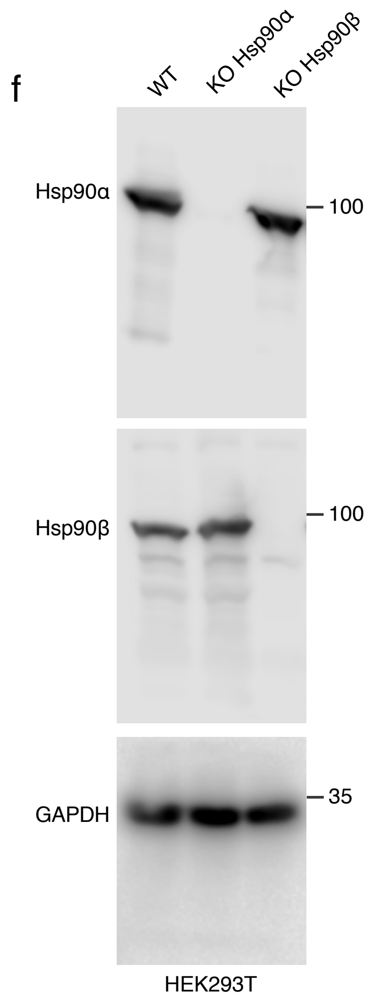

Supplementary figure 5

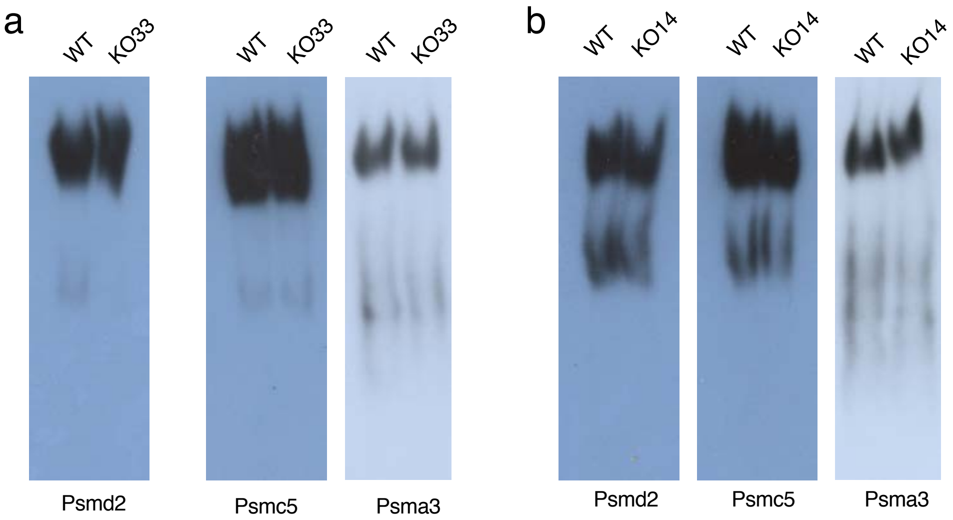

Supplementary figure 6

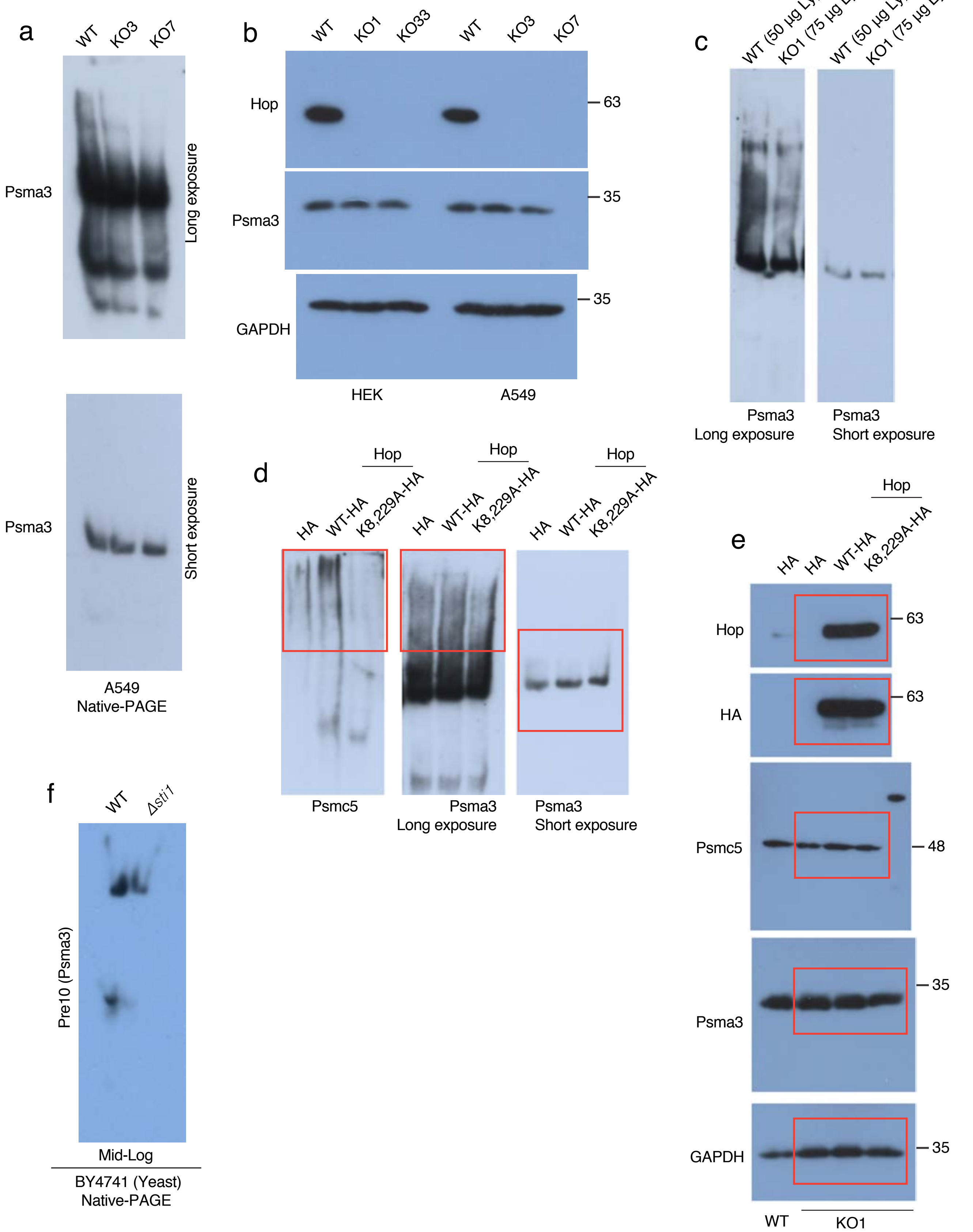

Supplementary figure 6

g

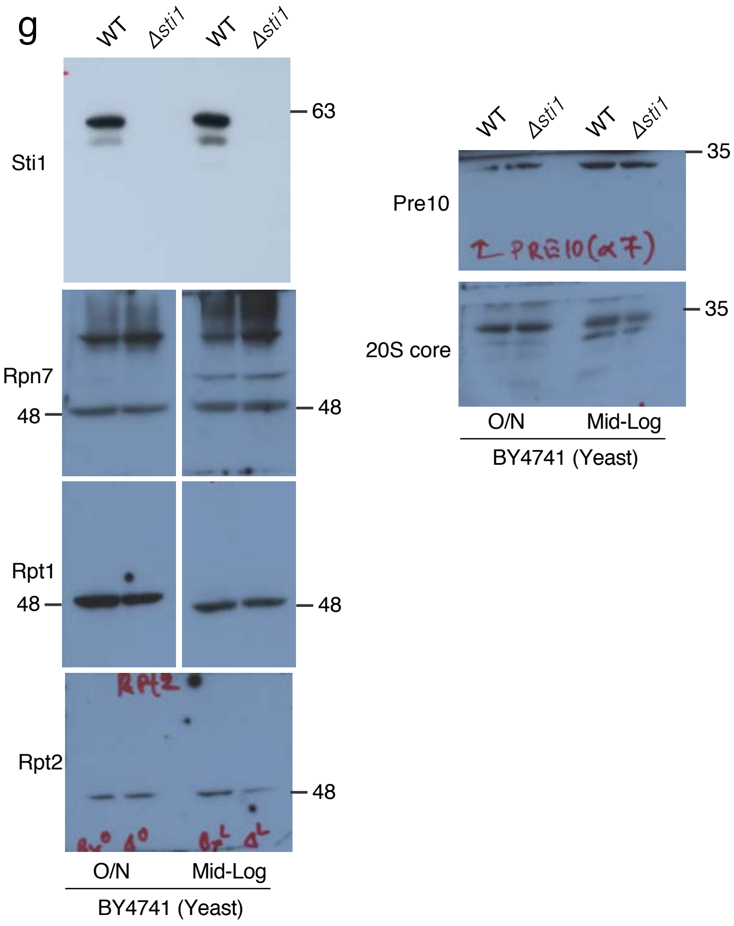

h

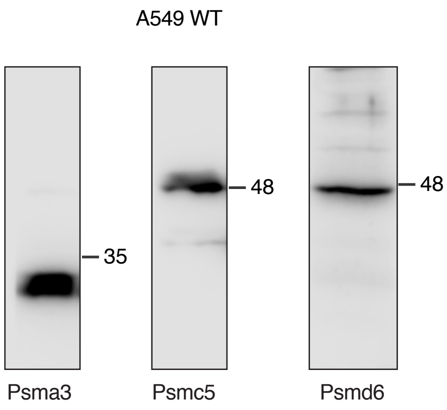

Supplementary figure 7

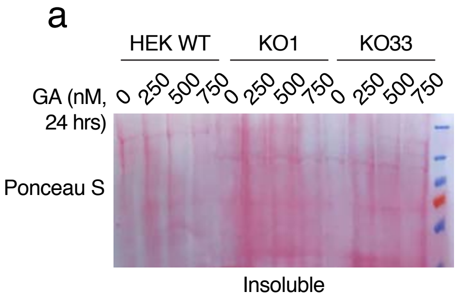

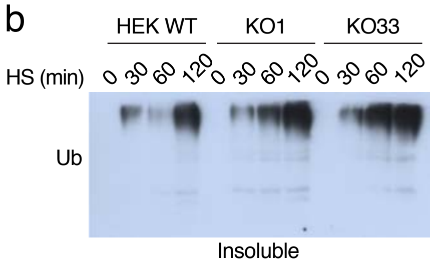

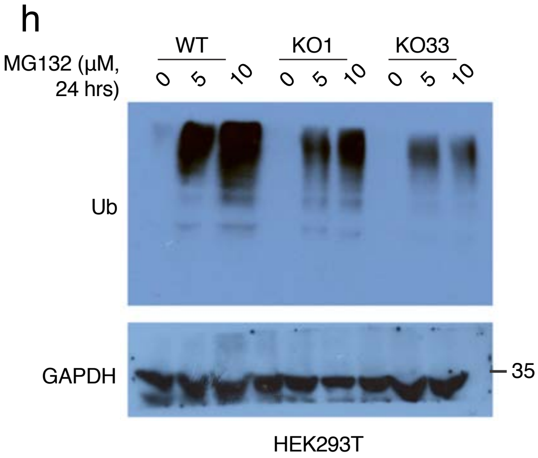

Supplementary figure 8

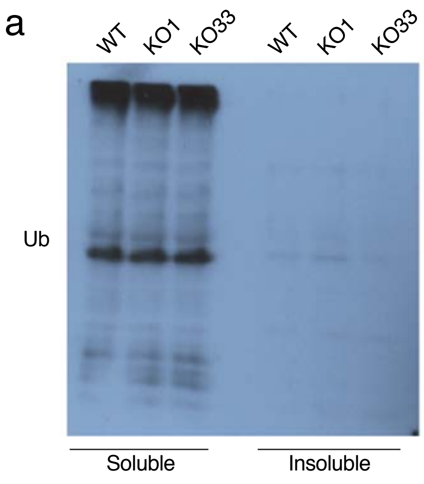

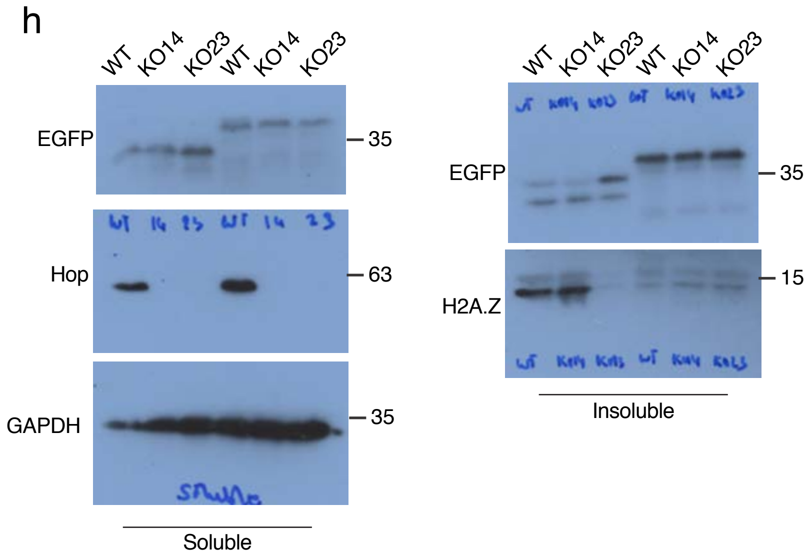

Supplementary figure 9

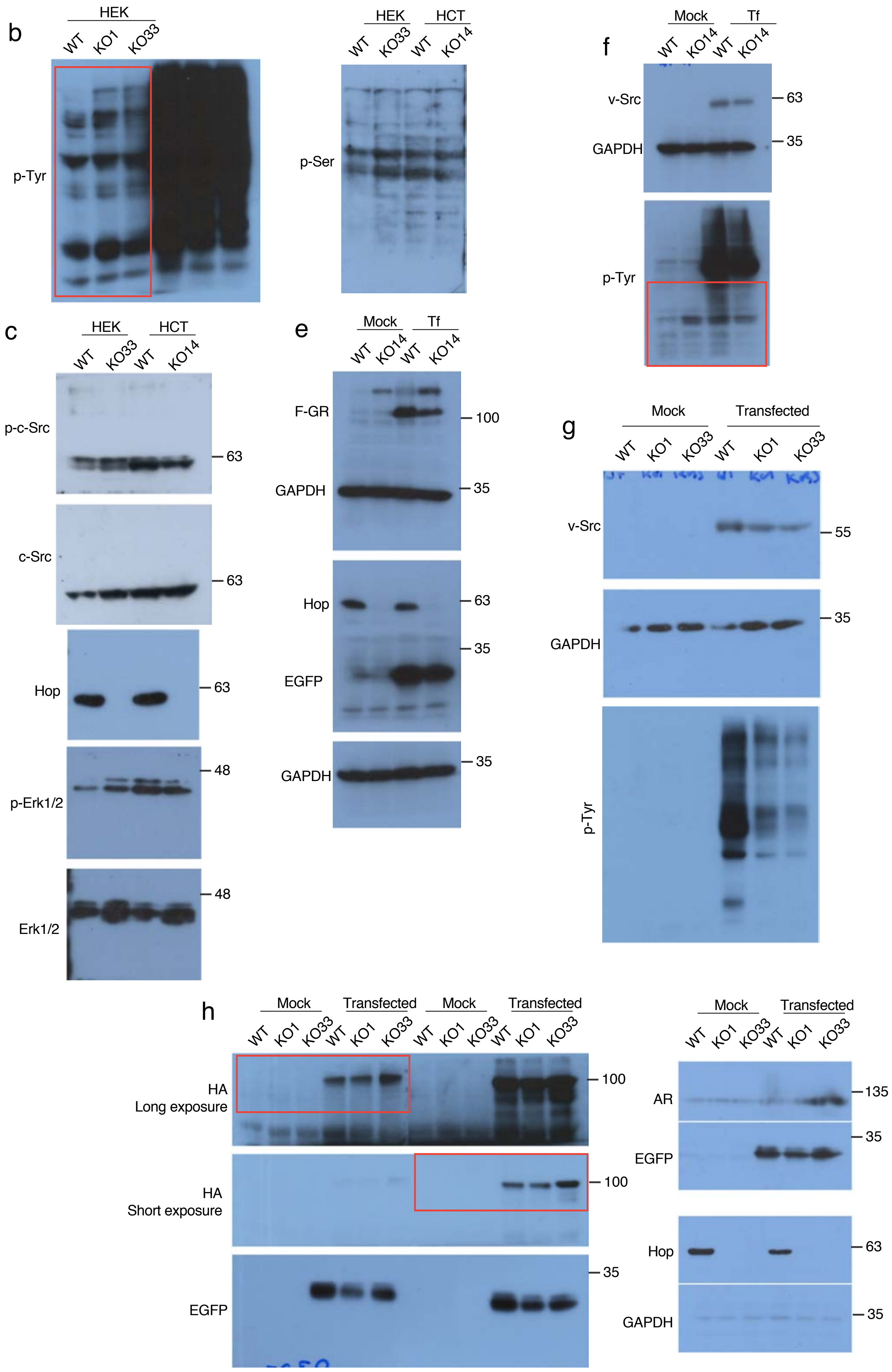

Supplementary figure 10

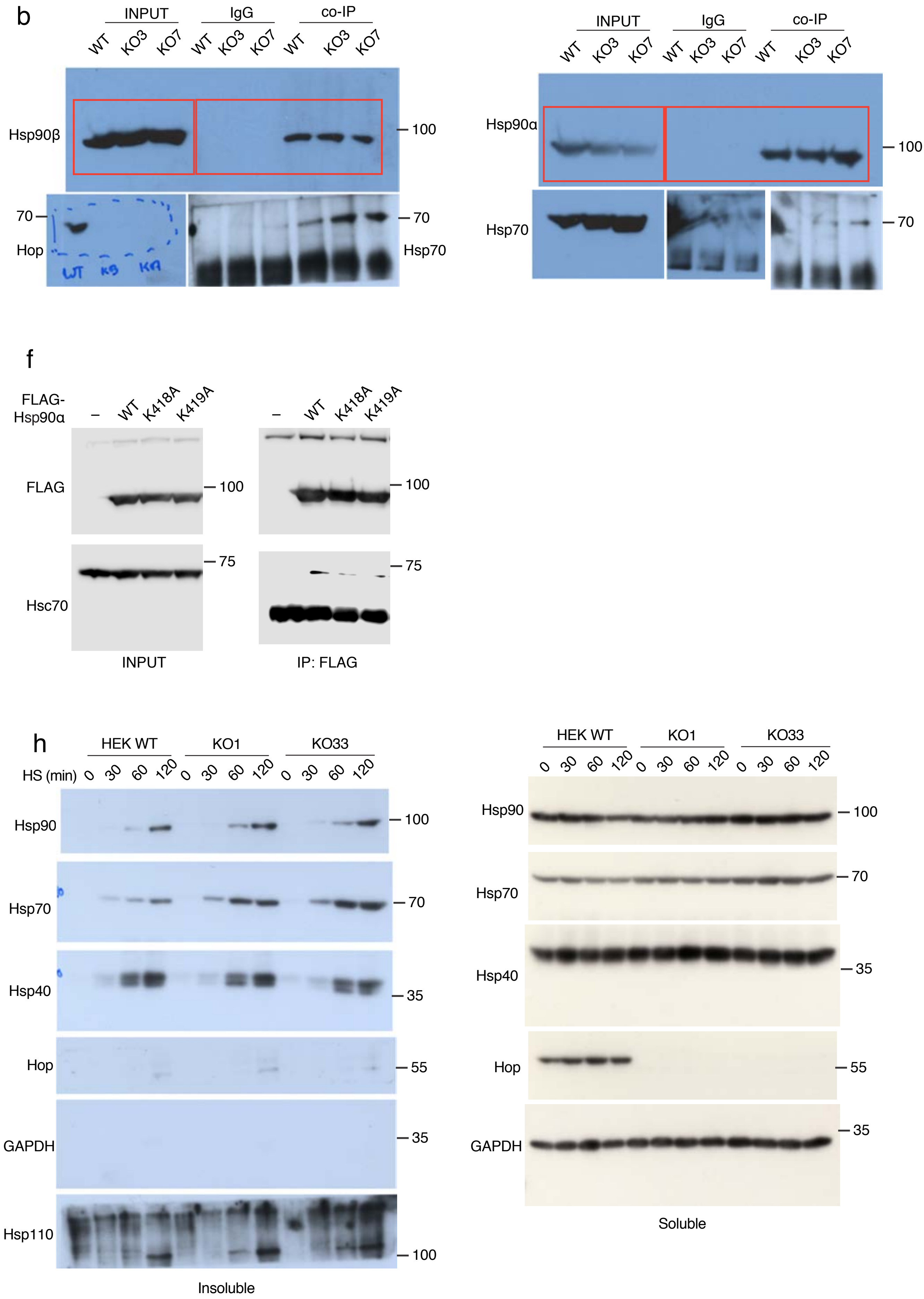

Supplement: Supplementary file 9 — Source Data [file 41467_2020_19783_MOESM9_ESM.zip › Source Data file/Uncropped images.pdf]
